# Curcumin as a complementary treatment in oncological therapy: a systematic review

**DOI:** 10.1007/s00228-024-03764-9

**Published:** 2024-10-19

**Authors:** Lisa C. Gutsche, Jennifer Dörfler, Jutta Hübner

**Affiliations:** Klinik für Innere Medizin II, Hämatologie und Internistische Onkologie, Universitätsklinikum Jena, Am Klinikum 1, Jena 07747 Germany

**Keywords:** Curcumin, Cancer, Complementary and alternative medicine (CAM), Nutritional supplements

## Abstract

**Purpose:**

Curcumin, the active ingredient in turmeric, is employed by numerous cancer patients to support conventional cancer therapy. This systematic review aims to summarize the existing clinical evidence and to provide an overview of the potential benefits and risks associated with curcumin supplementation.

**Methods:**

In January 2024, we conducted a systematic search of five electronic databases (Embase, Cochrane, PsycInfo, CINAHL, and Medline) using a complex search strategy. We included randomized controlled trials on the use, effectiveness, and potential harm of additional curcumin therapy in adult patients under cancer treatment. The risk of bias was assessed using Cochrane revised Risk of Bias Tool 2.0.

**Results:**

This systematic review included 34 randomized controlled trials involving 2580 patients out of 11143 search results. Included patients were primarily diagnosed with head and neck cancer, followed by breast, prostate, and colorectal cancer. Therapy concepts encompassed topical or systemic curcumin administration. The studies reported heterogeneous results concerning oral and skin symptoms, pain, weight alteration and changes in body composition, survival, and disease progression. Significant findings were reported for oral mucositis and weight loss. Considering risk of bias, all studies had moderate to high risk of bias. Regarding side effects, one study reported significantly more vomiting in the curcumin group.

**Conclusion:**

Although the results suggest promise in reducing mucositis and weight loss, a clear statement regarding the effectiveness of curcumin therapy on cancer patients cannot be made due to heterogeneous results and methodological limitations of the involved studies. Further investigations of higher quality are necessary to derive a definite recommendation for action.

**Supplementary Information:**

The online version contains supplementary material available at 10.1007/s00228-024-03764-9.

## Introduction

The natural polyphenol curcumin [1] is the most active constituent of the rhizome of the turmeric plant [2], *Curcuma longa* Linn. Found in many tropical and subtropical regions, *C. longa* belongs to the ginger family, *Zingiberaceae* [1]. The genus *Curcuma* contains about 120 species [2]. Apart from volatile oils and oleoresins [3], *C. longa* contains three different curcuminoids: 75–80% curcumin, 15–20% demethoxycurcumin, and 3–5% bisdemethoxycurcumin [4]. These are also present in other curcuma species, albeit in lower concentrations [5].

For thousands of years, curcumin has been used in traditional Chinese medicine and Ayurveda to combat inflammation and bacterial infections [1]. Curcumin's numerous properties, including anti-inflammatory, antioxidant, antibacterial, antiviral, antidiabetic, and wound-healing effects, make it an appealing subject of current pharmacological research. Multiple in vitro and in vivo studies showed its potential to induce apoptosis and inhibit cancer cell growth and metastasis through its effects on various signalling pathways. This includes modulation of NF-κB and STAT3, frequently overexpressed in head and neck tumor cells, the regulation of p53 protein in breast cancer cell lines as well as interference with cellular pathways in prostate cancer [3].

However, the pharmacokinetic properties of curcumin limit its clinical application. Poor solubility, low intestinal absorption rates, and rapid metabolism and elimination from the body result in low bioavailability [6]. Approaches for improvement include the combination of curcumin with additives like piperine, an active component of black pepper, which increases bioavailability by 2000%. Another possibility to improve bioavailability is developing curcumin into phospholipid complexes, liposomes, and nanoparticles [7].

The objective of this systematic review (SR) is to provide an overview of the current state of research on the clinical use of curcumin in various types of cancer. The aim is to cover a broad spectrum of results that could be relevant for cancer patients and to assess the efficacy of curcumin in its original or modified form, highlighting the beneficial and detrimental effects of curcumin in cancer therapy. This should facilitate the evaluation of possible clinical applications regarding potential benefits and risks for cancer patients in view of the current state of research.

## Method

### Criteria for including and excluding studies in the review

Inclusion and exclusion criteria are listed in Table 1 based on a PICO- model. Randomized controlled trials (RCTs) were included if they reported patient-relevant outcomes after treating mainly adult cancer patients (at least 80%) with interventions containing curcumin, either systemically or topically. All cancer types and stages were included due to the wide range of possible applications. Patient-relevant outcomes included symptoms recorded with validated instruments, body composition and weight alteration, side effects, interactions, mortality, morbidity, and patient reported outcomes including quality of life.

**Table 1:** Inclusion and exclusion criteria

PICO	Inclusion criteria	Exclusion criteria
Patient	- Majority cancer patients (all entities and stages): at least 80% cancer patients or defined subgroup with numerical data - Majority adults (at least 80% age > 18) or defined subgroups with numerical data - All genders - All ethnicities	- Patients with precancerous lesions or carcinoma in situ - Primary prevention - Preclinical studies
Intervention	- Interventions investigating the effect of curcumin application	- Multimodal interventions using curcumin to support another therapy, without separate evaluation of the individual interventions or where the effect of curcumin cannot be considered separately - Multipreparations - Studies in which no substance was administered, but only the level was measured
Comparison	- All possible control groups (placebo, standard care, observation)	- Other study types (one-armed/non-controlled studies, case report or series)
Outcome	- Primary endpoints were all patient-relevant symptoms or toxicities - Secondary endpoints were response data, survival data, and quality of life	- No patient- centered data: laboratory parameters without diagnosis (exception: established surrogates for patient-relevant outcomes: PSA, salivary cortisol for stress, antibodies after vaccination, blood pressure)
Others	- RCTs - Language: German and English - Full publication in a journal - Studies from 1995 onwards	- Grey literature (conference articles, abstracts, letters, ongoing studies, unpublished literature…) - Meta-analyses or reviews without systematic searches, without assessment of the risk of bias of the included studies or without evidence tables listing at least the populations, the interventions and control groups and the results of the included studies

We excluded studies without patient-relevant outcomes that reported only laboratory parameters without diagnosis, except for prostate-specific antigen (PSA). As PSA is considered an established surrogate parameter for tumor progression in prostate cancer according to the German S3 guideline on Complementary Oncology [8], we also included studies reporting PSA value changes. Studies with less than 80% adults or less than 80% cancer patients were excluded, as were studies on precancerous lesions or carcinoma in situ, multipreparations, studies without substance administration, and multimodal interventions without individual evaluation, where the effect of curcumin cannot be considered separately. Further criteria for rejecting studies were primary prevention, preclinical studies, grey literature, studies published before 1995, and study design other than RCT. Language restrictions were made to English and German. All methods were predefined within the methods of the German S3 guideline on Complementary Oncology [8].

### Study selection

In January 2024 a systematic search was conducted using five databases (Medline [Ovid], CINAHL [EBSCO], EMBASE [Ovid], Cochrane CENTRAL, and PsycINFO [EBSCO]). For each of these a complex search strategy was developed consisting of a combination of MeshTerms, keywords and text words in different spellings connected to cancer and curcumin therapy (Table 2). The search string was highly sensitive as it was not restricted by filters of study or publication type. After the first search, we assessed all reference lists of the included RCTs and of SRs found in first search for publications not found previously and assessed their suitability according to the predefined inclusion and exclusion criteria. The search results were imported into EndNote 20 and duplicates were removed. Two reviewers (LG, JD) conducted the title-abstract screening independently. In case of disagreement, consensus was reached through discussion or consultation with a third reviewer (JH). Subsequently, both reviewers retrieved and independently screened all full texts. If the title and abstract did not provide sufficient information for screening, a full-text copy was retrieved. Furthermore, the bibliography lists of all retrieved articles were searched for relevant studies.

**Table 2:** Search strategy

Data base	Search string
Ovid Medline	**1** curcumin$.mp. or exp Curcumin/ or turmeric.mp. or curcuma.mp **2** exp neoplasms/ or neoplasm$.mp or cancer$.mp. or tumo?r$.mp. or malignan$.mp. or oncolog$.mp. or carcinom$.mp. or leuk?emia.mp. or lymphom$.mp. or sarcom$.mp **3** 1 AND 2 **4** limit 3 to english or limit 3 to german **5** (4 and humans/) or (4 not animals/) **6** ((((comprehensive* or integrative or systematic*) adj3 (bibliographic* or review* or literature)) or (meta-analy* or metaanaly* or "research synthesis" or ((information or data) adj3 synthesis) or (data adj2 extract*))).ti,ab. or (cinahl or (cochrane adj3 trial*) or embase or medline or psyclit or (psycinfo not "psycinfo database") or pubmed or scopus or "sociological abstracts" or "web of science" or central).ab. or ("cochrane database of systematic reviews" or evidence report technology assessment or evidence report technology assessment summary).jn. or Evidence Report: Technology Assessment*.jn. or (network adj1 analy*).ti,ab.) or (((review adj5 (rationale or evidence)).ti,ab. and review.pt.) or meta-analysis as topic/ or Meta-Analysis.pt.) **7** Randomi?ed controlled trial?.pt. or controlled clinical trial?.pt. or randomi?ed.ti,ab.or placebo.ti,ab. or drug therapy.sh. or randomly.ti,ab. or trial?.ti,ab. or group?.ti,ab **8** 5 AND (6 OR 7)
Ovid Embase	**1** curcumin$.ti,ab. or exp Curcumin/ or turmeric.ti,ab. or curcuma.ti,ab **2** exp neoplasm/ or neoplasm$.mp or cancer$.mp. or tumo?r$.mp. or malignan$.mp. or oncolog$.mp. or carcinom$.mp. or leuk?emia.mp. or lymphom$.mp. or sarcom$.mp **3** 1 AND 2 **4** limit 3 to english or limit 3 to german **5** (4 and humans/) or (4 not animals/) **6** ((((comprehensive* or integrative or systematic*) adj3 (bibliographic* or review* or literature)) or (meta-analy* or metaanaly* or "research synthesis" or ((information or data) adj3 synthesis) or (data adj2 extract*))).ti,ab. or (cinahl or (cochrane adj3 trial*) or embase or medline or psyclit or (psycinfo not "psycinfo database") or pubmed or scopus or "sociological abstracts" or "web of science" or central).ab. or ("cochrane database of systematic reviews" or evidence report technology assessment or evidence report technology assessment summary).jn. or Evidence Report: Technology Assessment*.jn. or (network adj1 analy*).ti,ab.) or (exp Meta Analysis/ or ((data extraction.ab. or selection criteria.ab.) and review.pt.)) **7** crossover procedure/ or double blind procedure/ or randomized controlled trial/ or single blind procedure/ or (random$ or factorial$ or crossover$ or (cross adj1 over$) or placebo$ or (doubl$ adj1 blind$) or (singl$ adj1 blind$) or assign$ or allocat$ or volunteer$).ti,ab,de **8** 5 AND (6 OR 7)
Cochrane	**#1** [mh curcumin] or curcumin* or turmeric or curcuma **#2** [mh neoplasms] or neoplasm* or cancer? or tum*r? or malignan* or oncolog* or carcinom* or leuk*mia or lymphoma? or sarcoma? **#3 #**1 AND #2
Ebsco—PsychINFO	**S1** TX curcumin* OR TX turmeric OR TX curcuma **S2** ((DE "Neoplasms" OR DE "Benign Neoplasms" OR DE "Breast Neoplasms" OR DE "Endocrine Neoplasms" OR DE "Leukemias" OR DE "Melanoma" OR DE "Metastasis" OR DE "Nervous System Neoplasms" OR DE "Terminal Cancer") OR (TX neoplasm* OR TX cancer OR TX tumo#r OR TX malignan* OR DE „oncology “ OR TX oncolog* OR TX carcinom* OR TX leuk#emia OR TX lymphoma OR TX sarcoma)) **S3** (LA German OR LA English) **S4** S1 AND S2 AND S3 **S5** ((comprehensive* OR integrative OR systematic*) N3 (bibliographic* OR review* OR literature)) OR (meta-analy* or metaanaly* or "research synthesis" OR ((information OR data) N3 synthesis) OR (data N2 extract*)) OR ((review N5 (rationale OR evidence)) AND DE "Literature Review") OR (AB(cinahl OR (cochrane N3 trial*) OR embase OR medline OR psyclit OR pubmed OR scopus OR "sociological abstracts" OR "web of science" OR central)) OR DE "Meta Analysis" OR (network N1 analy*) **S6** DE "Treatment Effectiveness Evaluation" OR DE "Treatment Outcomes" OR DE "Psychotherapeutic Outcomes" OR DE "Placebo" or DE "Followup Studies" OR placebo* OR random* OR "comparative stud*" OR (clinical N3 trial*) OR (research N3 design) OR (evaluat* N3 stud*) OR (prospectiv* N3 stud*) OR ((singl* OR doubl* OR trebl* OR tripl*) N3 (blind* OR mask*) **S7** S4 AND (S5 OR S6)
Ebsco- CINAHL	**S1** MH “curcumin” OR TX curcumin* OR TX turmeric OR TX curcuma **S2** (MH "Neoplasms + " OR TX neoplasm* OR TX cancer OR TX tumo#r OR TX malignan* OR TX oncolog* OR TX carcinom* OR TX leuk#emia OR TX lymphoma OR TX sarcoma) **S3** (LA German OR LA English) **S4** S1 AND S2 AND S3 **S5** (TI (systematic* n3 review*)) or (AB (systematic* n3 review*)) or (TI (systematic* n3 bibliographic*)) or (AB (systematic* n3 bibliographic*)) or (TI (systematic* n3 literature)) or (AB (systematic* n3 literature)) or (TI (comprehensive* n3 literature)) or (AB (comprehensive* n3 literature)) or (TI (comprehensive* n3 bibliographic*)) or (AB (comprehensive* n3 bibliographic*)) or (TI (integrative n3 review)) or (AB (integrative n3 review)) or (JN “Cochrane Database of Systematic Reviews”) or (TI (information n2 synthesis)) or (TI (data n2 synthesis)) or (AB (information n2 synthesis)) or (AB (data n2 synthesis)) or (TI (data n2 extract*)) or (AB (data n2 extract*)) or (TI (medline or pubmed or psyclit or cinahl or (psycinfo not “psycinfo database”) or “web of science” or scopus or embase)) or (AB (medline or pubmed or psyclit or cinahl or (psycinfo not “psycinfo database”) or “web of science” or scopus or embase or central)) or (MH “Systematic Review”) or (MH “Meta Analysis”) or (TI (meta-analy* or metaanaly*)) or (AB (meta-analy* or metaanaly*)) or network n1 analy* **S6** (MH "Clinical Trials + ") or PT Clinical trial or TX clinic* n1 trial* or TX ( (singl* n1 blind*) or (singl* n1 mask*)) or TX ((doubl* n1 blind*) or (doubl* n1 mask*)) or TX ( (tripl* n1 blind*) or (tripl* n1 mask*)) or TX ((trebl* n1 blind*) or (trebl* n1 mask*)) or TX randomi* control* trial* or (MH "Random Assignment") or TX random* allocat* or TX placebo* or MH "Placebos") or MH "Quantitative Studies") or TX allocat* random* **S7** S4 AND (S5 OR S6)

### Assessment of risk of bias and methodological quality

The included studies' risk of bias was analyzed using the Cochrane revised Risk of Bias Tool 2.0. [9]. Additional criteria regarding methodology included population size, use of power analysis, appropriate statistical tests (e.g. controlling for premises or multiple testing), and selective outcome reporting (reporting all assessed outcomes with specification of statistical data such as *p*-values). Two reviewers (LG, JD) assessed all characteristics independently. In case of disagreement, a third reviewer (JH) was consulted, and consensus was reached through discussion.

### Data extraction

Data extraction was performed by one reviewer (LG) and controlled by two reviewers independently (JD, JH). We have summarized the main characteristics and results of the included studies in tabular form. As a template for data extraction, standardized evidence tables from the national Guideline on Complementary and Alternative Medicine in Oncological Patients of the German Guideline Program in Oncology [8] were used. The tables displaying the data extracted from the included studies are presented below (Table 3).

**Table 3:** Characteristics of included studies

Reference	Study type	n/ cancer type/ Dropout	Intervention/ Duration	Endpoints	Outcomes
**Arun et al. (2020)**	RCT	Head and neck cancer Prior RTx + CTx and/or surgery, RTx or RCTx during intervention Included: *n* = 64 Analyzed: *n* = 61 A: *n* = 30 B: *n* = 31 Dropout-rate: overall 4.7% (unknown group allocation)	Arm A: 500 mg turmeric extract capsules/day (BCM-95®/Curcugreen®) Arm B: Placebo Duration: Until completion of RTx or RCTx treatment (approx. 4 weeks)	T0: Baseline T1: Day 7 T2: Day 14 T3: Day 21 T4: Day 28 Follow-up: 2 months after treatment 1. Severity of oral mucositis (Objective WHO oral mucositis scale, Subjective NCI CTCAE assessment, version 4.0)	1. Objective scale: sign. difference at T3 (*p* < 0.001), T4 (*p* < 0.001) and follow-up (after 2 months) (*p* < 0.001); ns at other time points; 2. subjective scale: sign. difference at T3 (*p* < 0.001), T4 (*p* < 0.001) and follow-up (*p* < 0.001); ns at other time points
**Chaiworra** **mukkul et al. (2022)**	RCT	Solid cancer Concomitant CTx or targeted therapy or RTx with palliative intent Included: *n* = 33 Analyzed: *n* = 25 A: *n* = 17 B: *n* = 16 Dropout-rate: overall 24% (unknown group allocation)	Arm A: 800 mg curcumin twice daily orally (400 mg per caplet, contained 240 mg curcuminoids) Arm B: Placebo (corn starch) Duration: 8 weeks	T0: Baseline T1: Week 4 (Safety Assessment) T2: Week 8 1. Body tissue composition (body fat mass, skeletal muscle mass, percentage of body fat) 2. Body weight 3. Body Mass Index (BMI) 4. Hand grip muscle strengthening 5. Basal metabolic rate	1. Change of body fat mass in kg (SEM): A: -1.25 (0.87), B: + 0.63 (0.55), *p* = 0.119, ns; 2. skeletal muscle mass in kg (SEM): A: -0.35 (0.60), B: + 0.33 (0.42), *p* = 0.408, ns; 3. percentage of body fat (SEM): A: -0.47 (0.95), B: -0.29 (0.82), *p* = 0.893, ns; 4. Weight loss in kg (SEM): A: -1.4 (0.89), B: -1.12 (0.73), *p* = 0.810 ns 5. BMI change (SEM): A: -0.50 (0.31), B: -0.44 (0.26), *p* = 0.886 ns; 6. Change of hand grip muscle strength in kg (SEM): right-sided: A: -2.47 (1.46), B: -5.36 (2.54), *p* = 0.318; left-sided: A: -1.98 (1.24), B: -5.43 (3.11), *p* = 0.317 ns 7. Basal metabolic rate in kcal (SEM): A: -13.47 (21.94), B: + 15.30 (13.76), *p* = 0.336 ns
**Charantimath (2016)**	RCT	Head and neck cancer RCTx during intervention Included: *n* = 40 Analyzed: *n* = 40 A: *n* = 20 B: *n* = 20 Dropout-rate: overall 0% / no information	Arm A: Curcumin oral gel (Cure next, Abbott Pharmaceuticals) 10 mg C. longa extract/g gel, 3 times a day after meals Arm B: Chlorhexidine gluconate 1.0% Duration: 2 weeks	T0: Baseline T1: After 1 week T2: After 2 weeks 1. Pain (NRS) 2. Erythema (Oral Mucositis Assessment scale) 3. Ulceration (Oral Mucositis Assessment scale) 4. Mucositis (WHO mucositis scale)	1. Sign. difference between T0 and T2: A: 4.18 (1.13), B: 1.89 (0.68), *p* = 0.0001 2. Sign. difference between T0 and T2: A: 1.76 (1.13), B: 0.83 (0.68), *p* = 0.0006 3. Sign. difference between T0 and T2: A: 1.76 (1.13); B: 0.83 (0.68); *p* = 0.0001 4. Sign. difference between T0 and T2: A: 1.76 (1.13); B: 0.83 (0.68); *p* = 0.0001 Reported results partly inconclusive
**Choi et al. (2019)**	RCT	Prostate cancer Prior IAD Included: *n* = 97 Analyzed: *n* = 82 A: *n* = 49 B: *n* = 48 Dropout-rate: overall 17.5%, A: 22.4%, B: 12.5%	Arm A: 1440 mg Curcumin capsules/day Arm B: Placebo Duration: 6 months	T0: Baseline T1-T6: Each month Follow-up: every 3 months until ADT was required again or up to 36 months 1. Duration of off- treatment in months, defined as period from start of ADT withdrawal until restart of ADT 2. Change from baseline in the PSA and testosterone levels during the 6 months of the curcumin treatment period 3. PSA progression rate at 6 months 4. Health-related quality of life (HRQOL) scores at 6 months: Functional Assessment of Cancer Therapy-Prostate (FACT-P) questionnaire, International Prostate Symptom Score (IPSS) questionnaire, International Index of Erectile Function (IIEF-15) questionnaire 5. Safety measurements Subgroup analysis by reason for ADT: Metastatic prostate cancer vs biochemical recurrence (BCR)	1. Median (95% CI) A: 16.3 (12.3–20.3); B:18.5 (12.5–23), ns (*p* = 0.4816); 2. subgroup analysis: metastatic prostate cancer 18.6 vs 19.3, *p* = 0.824; BCR 12.8 vs 14.2, *p* = 1.0000, ns 3. Changes of PSA level (ng/ml) A: 0.52 (0.07–1.06), B: 0.32 (0.08–2.85), ns (*p* = 0.7592) 4. PSA progression significantly lower in A; A: 10.3%, B: 30.2%, *p* = 0.0259; 5. subgroup analysis: metastatic prostate cancer 10.5% vs 42.1%, *p* = 0.0542; BCR 10.0% vs 20.8%, *p* = 0.8560, ns 6. FACT-P: A: 113.3 (98.0–138.6), B: 115.2 (103.5–135.0), *p* = 0.7401 7. IPSS total: A: 12.0 (7.0–18.0), B: 12.5 (11.0–17.0), *p* = 0.8125; 8. IPSS QoL: A: 2.0 (2.0–3.0), B: 2.0 (2.0–3.0), *p* = 0.3857; 9. IIEF-15: A: 5.0 (5.0–7.0), B: 5.0 (5.0–7.0), *p* = 0.8035 10. Patients with AEs: A: 15.6%, B: 34.8%, sign. difference (*p* = 0.0349)
**Delavarian et al. (2019)**	RCT	Head and neck cancer RTx during intervention Included: *n* = 32 Analyzed: *n* = 29 A: *n* = 16 B: *n* = 16 Dropout-rate: overall 9.4%, A: 6.3%, B: 12.5%	Arm A: 80 mg/day oral nanocurcumin (1 capsule of SinaCurcumin® 80 per day, contains 80 mg of curcumin-loaded nanomicelles) Arm B: Placebo (lactose) Duration: 6 weeks	T0: Baseline T1-T6: Day 7, 14, 21, 28, 35, 42 of RTx 1. Mucositis (NCI-CTC v.2) 2. Weight change	1. Mean (SD): 2. T1: A: 0, B: 0.37 (0.50), *p* = 0.007; 3. T2: A: 0.62 (0.80), B: 1.5 (0.51), *p* = 0.002; 4. T3: A: 1.31 (0.47), B: 2.37 (0.71), *p* < 0.001; 5. T4: A: 2.00 (0.84), B: 2.60 (0.63), *p* = 0.011; 6. T5: A: 2.26 (0.88), B: 3.21 (0.69), *p* = 0.006; 7. T6: A: 2.40 (0.91), B: 3.35 (0.74), *p* = 0.005; sign. differences 8. Weight change in kg (SD): A: 0.43 (0.81), B: 1.32 (0.87), *p* = 0.003, sign. difference
**Fardad et al. (2022)**	RCT	Various cancers CTx during intervention Included: *n* = 75 Analyzed: *n* = 71 A: *n* = 25 B: *n* = 25 C: *n* = 25 Dropout-rate: overall 5.3%, A: 8%, B: 4%, C: 4%	Arm A: Curcumin gel 0.5%, four times daily; entire mouth covered with thin layer of gel using cotton applicator Arm B: Mucosamin® oral mucosal spray (Professional Dietetics®, Italy), four puffs daily Arm C: Commercially available oral chlorhexidine mouthrinse 0.2% (Behsa®, Iran), in 1:1 dilution for 1 min four times a day Duration: 2 weeks	T0: Baseline T1-T14: Daily, for 2 weeks 1. Mucositis: onset of complete recovery after starting the treatment (WHO Oral Toxicity Scale, Oral Mucositis Assessment Scale, NRS) 2. Drug safety	1. WHO: day 3: A: 1.04, B: 1.42, C: 1.46, *p* = 0.05, according to authors statistically sign.; 2. in A full recovery from T4, no complete recovery in B and C during study; 3. OMAS: day 3, erythema: A: 0.48, B: 1.04, C: 0.88, *p* = 0.003, statistically sign.; 4. full recovery: A from day 4, B from day 6, no complete recovery in C; 5. OMAS day 5, ulceration: A: 0.00, B: 0.29, C: 0.42, *p* = 0.04, statistically sign.; 6. full recovery: A from day 5, B from day 11, no complete recovery in C; 7. NRS day 3: A: 0.78, B: 2.50, C: 1.79, *p* = 0.02, statistically sign.; 8. full recovery: A from day 6, B from day 11, C from day 14 9. No adverse reactions related to all products, except for eight patients who experienced a stinging sensation in C
**Gunther et al. (2022)**	RCT	Rectal cancer RCTx during intervention Included: *n* = 22 Analyzed: *n* = 22 (ITT) A: *n* = 15 B: *n* = 7 Dropout-rate: overall 0% (but one patient did not undergo surgery)	Arm A: Curcumin C3 Complex (Sabinsa Corporation) 4 g orally twice daily, approx. 1 h before RTx Arm B: Placebo Duration: Until the end of RTx treatment + 6 weeks	T0: Baseline T1-T6: Toxicity check (CTCAE) weekly during RCTx, T7: Toxicity check 1 month after completion of RCTx and 6 weeks of maintenance therapy, surgery usually within 1–3 weeks from this toxicity check Follow-up: until 2021 1. Surgical pCR rate 2. Downstaging at time of surgery 3. Local control (LC)/ time to local regional failure (TTLRF)/ time to distant failure (TTDF) 4. PFS 5. OS + Curcumin concentration	1. pCR n(%) at time of surgery: A: 1 (7%), B: 2 (33%), *p* = 0.18, ns 2. n (%): A: 7 (47%), B: 4 (66%), *p* = 0.64, ns; ns in terms of pathologic tumor stage (*p* = 0.34) or tumor regression grade (*p* = 0.44) 3. 5-year cumulative incidence of local regional failure (95%CI): A: 6.7% (0, 19.7%), B: 14.3% (0, 42%), *p* = 0.55, ns; 4. 5-year cumulative incidence of distant failure (95%CI): A: 33.3% (8.5, 58.2%), B: 28.6% (0, 65.0%), *p* = 0.97, ns 5. 5-year PFS (95%CI): A: 66.7% (46.6, 95.3%), B: 71.4% (44.7, 100%), *p* = 0.80, ns 6. 5-year OS (95%CI): A: 85.7% (69.2, 100%), B: 85.7% (63.3, 100%), *p* = 0.70, ns **Curcumin concentration:** median serum curcumin concentration before (range): 3.04 ng/ml (1.24–18.88 ng/ml), 1 h after: 3.32 ng/ml (0.84–5.36 ng/ml), *p* = 0.33, ns, increased: *n* = 4, decreased or remained constant: *n* = 8; median tissue curcumin concentration: A: 33.7 ng/mg (0.1–4,765.7 ng/mg), B: 0.0 ng/mg tissue (0.0–0.0 ng/mg); no association between tissue curcumin concentration and TTLRF (*p* = 0.23) or pCR (*p* = 0.17)
**Hejazi et al. (2013 and 2016)**	RCT	Prostate cancer RTx (EBRT), in combination with hormone ablation during intervention Included: *n* = 45 Analyzed: *n* = 45 (ITT) A: *n* = 20 B: *n* = 20 Dropout-rate: overall 11.1%, A: 10%, B: 15%	Arm A: 3000 mg Curcumin capsules/day Arm B: Placebo Duration: Until the end of RTx treatment (approx. 9 weeks)	T0: 1 week prior to RTx onset T1: 3 months after treatment 1. PFS (within 1 year) 2. QoL (EORTC QLQ-PR25)	1. PSA level reduction (ng/ml) within 3 months: mean change (SD) A: 0.12 (0.16), B: 0.13 (0.06), *p* = 0.78; no information on PFS within 1 year 2. Mean change A-B 3 months after end of RTx (95% CI); 3. urine symptoms: -14.1 (-24.7, -3.4), *p* = 0.011, statistically sign. fewer symptoms in A; 4. bladder symptoms: 5.4 (-4.5, 15.4), *p* = 0.275; 5. treatment related symptoms: 7.9 (-3.1, 18.9), *p* = 0.155; 6. sexual activity: -3.5 (-19.0, 12.0), *p* = 0.652
**Howells et al. (2019)**	RCT	Colorectal cancer CTx during intervention Included: *n* = 28 Analyzed: *n* = 27 (ITT, 1 subsequently deemed ineligible, PP: 24) A: *n* = 18 B: *n* = 9 Dropout-rate: overall 11.1% A:16.7% B: 0%	Arm A: FOLFOX ± Bevacizumab plus 2 g Curcumin C3 Complex/d oral (Sabinsa Corp, approx. 80% Curcumin and 20% Demethoxycurcumin and Bisdemethoxycurcumin) (CUFOX) Arm B: FOLFOX ± Bevacizumab (FOLFOX) Duration: Once every 2 weeks for ≤ 12 cycles or until disease progression, unacceptable toxicity, death, or withdrawal	T0: Baseline T1-T4: 1st, 3rd, 5th, 24th week 1. Safety and tolerability (CTCAE), incl. neurotoxicity 2. Efficacy via progression-free survival (PFS), objective response rate (ORR), overall survival (OS) 3. Quality of life (Quality of Life Questionnaire-C30) 4. Plasma curcuminoids and C-X-C-motif chemokine ligand 1 (CXCL1)	1. Cycles median (mean, range): A: 12 (9.4; 0–12), B: 3 (5.1; 0–12); 3 patients with 25% dose reduction of 5-FU/Oxaliplatin in A; 1 participant stopped curcumin early owing to nausea; 2. total AEs: A: 282, B: 103; 3. serious AEs per cycle: A: 0.3, B: 0.7, *p* = 0.521, ns; 4. AEs per cycle: A: 2.1, B: 2.4, *p* = 0.142, ns; most common: grade 1 or 2 fatigue and peripheral neuropathy, grade 3 or 4 – thromboembolic events (3 in A); 5. AEs with causality possibly or probably related to curcumin: primarily gastrointestinal, most common diarrhea, positive correlation between overall neurotoxicity scores and number of CTx cycles (Pearson’s correlation coefficient = 0.429, *p* < 0.001), ns in neurotoxicity scores between arms at cycles 6 (*p* = 0.223) or 12 (*p* = 0.204) 6. 22 died during follow up period: A: 13 of 18, B: 9 of 9; 2 did not commence owing to rapid disease progression (1 in A, 1 in B), 4 proceeded to surgical resection of liver metastases (2 in A, 2 in B) between cycles 7 and 12; 7. ITT: PFS: HR 0.571 (95% CI: 0.24, 1.36; *p* = 0.200), ns; 8. OS: HR 0.339 (95% KI 0.141, 0.815; *p* = 0.016), statistically sign.; 9. PP (A *n* = 15, B *n* = 9): median (range): 10. PFS: A: 320d (175-405d), B: 171d (9-214d), 6-months-survival (95% CI): A 73.3% (43.6%, 89.1%), B: 33.3% (7.8%, 62.3%); PFS HR: 0.549 (95% KI 0.225, 1.34, *p* = 0.183), ns; 11. median OS (range/CI): A: 596d (323d-still alive), 93.3% (61.3%, 99.0%), B: 200d (9-563d), 55.6% (20.4%, 80.5%); OS HR: 0.271 (95% KI: 0.106, 0.697, *p* = 0.004), statistically sign.; 12. ORR at cycle 6: A: 66.7%, B: 44.4%, *p* = 0.285, ns; 13. cycle 12: A: 53.3%, B: 11.1%, *p* = 0.039, statistically sign., 14. no complete responses observed; 15. T1: stable disease: A: 28%, B: 22%, *p* = 0.756, partial response: A: 56%, B: 44%, *p* = 0.586, ns 2. Subgroup analysis: excluding patients who proceeded to surgical resection (A *n* = 15, B *n* = 7), HR (95% CI, p): 3. PFS: 0.152 (0.046, 0.501, *p* < 0.001); 4. OS: 0.146 (0.047, 0.459, *p* < 0.001); 5. statistically sign. differences; 6. ORR 6 months: A: 50.0%, B: 28.6%, *p* = 0.340, ns 7. ns (*p* = 0.248) 8. Two patients with plasma curcumin glucuronide concentrations < 1.00 pmol/ml were not included in PP analyses; 9. CXCL1 concentration ns (*p* = 0.712)
**Kia et al. (2021)**	RCT	Various cancers CTx ± RTx during intervention Included: *n* = 50 Analyzed: *n* = 50 A: *n* = 25 B: *n* = 25 Dropout-rate: overall 0%/ no information	Arm A: 80 mg nanomicelle Curcumin capsules (SinaCurcumin, containing curcumin, bisdemethoxycurcumin, and desmethoxycurcumin) twice a day Arm B: Placebo (mostly made of sugar) Duration: 7 weeks	T0: Start of CTx or RCTx T1-T4: week 1, 2, 4, 7 1. Mucositis (WHO Mucositis Scale) 2. Pain (NRS)	1. WHO grade mean (SD): 2. T1: A: 0.36 (0.49), B: 0.72 (0.46), *p* = 0.010, statistically sign.; 3. T2: A: 1.00 (0.64), B: 1.28 (0.46), *p* = 0.083, ns; 4. T3: A: 1.44 (0.58), B: 1.88 (0.73), *p* = 0.022, statistically sign.; 5. T4: A: 1.36 (0.64), B: 2.20 (0.71), *p* < 0.001, statistically sign 6. NRS mean (SD): 7. T1: A: 0.68 (1.11), B: 0.64 (1.03), *p* = 0.896, ns; 8. T2: A: 1.36 (1.91), B: 1.84 (1.57), *p* = 0.337, ns; 9. T3: A: 2.44 (2.00), B: 3.28 (1.57), *p* = 0.105, ns; 10. T4: A: 2.64 (2.04), B: 4.44 (1.68), *p* = 0.001, statistically sign Subgroup analysis: CTx only: 1. Statistically sign. difference in T1 (*p* < 0.001), T2 (*p* = 0.006), T3 (*p* = 0.008), T4 (*p* < 0.001); 2. Statistically sign. difference in T2 (*p* = 0.004), T3 (*p* = 0.005), T4 (*p* < 0.001), T1 ns; CTx and RTx (*n* = 13): 1. Statistically sign. difference in T3 (*p* = 0.009), T4 (*p* = 0.012), T1 and T2 ns; 2. ns
**Kumar et al. (2016)**	RCT	Head and neck cancer RCTx (EBRT) during intervention Included: *n* = 73 Analyzed: *n* = 60 A: *n* = 38 B: *n* = 35 Dropout-rate: overall 17.8% A: 21.1%, B: 14.3%	Arm A: EBRT + weekly 40 mg/m^2^ cisplatin i.v. + curcumin 2 capsules 1 g daily every 8 h Arm B: EBRT + weekly 40 mg/m^2^ cisplatin i.v Duration: 7 to 9 weeks	T0: Baseline from T1: Weekly during therapy, Follow-up: monthly until 1 year 1. Tumor and nodal response (WHO response criteria) 2. Acute and late RTx toxicity (WHO toxicity criteria, RTOG Acute Radiation Morbidity Scoring Criteria) 3. Subgroup analysis on numerous prognostic indicators	1. Response after 1 year: no evidence of disease: A: 66.7%, B: 56.7%; residual disease: A: 16.66%, B: 26.7%; reccurent disease: A: 10%, B: 13.3%; progressive disease: A: 6.7%, B: 3.33%, without given *p*-values; mean follow-up duration (range): 18 months (6–20), all at least 12 months 2. Acute skin toxicity grade III: A: 3.3%, B: 20%; mucosal toxicity: A: 10%, B: 20%; upper GI toxicity grade 3 and 4: A: 20% and 6.7%, respectively, B: 6.7% and 0%, respectively; grade 2 hemoglobin toxicity: A: 2 (6.66%), B: 3 (10%), *p* = 0.300, ns; leukopenia grade 1: *p* = 0.313; leukopenia grade 2: A: 1 (3.33%), B: 4 (13.33%); leukopenia grade 3: A: 1 (3.3%), B: 1 (3.3%); blood urea toxicity grade 1: A: 0, B: 5 (16.66%), *p* = 0.019, stat. sign.; 3. stat. sign. less grade 3 and 4 vomiting (*p* = 0.037) in B; treatment completed after 7.3 weeks: A: 90%, B: 80%; in B at 20% delay due to grade 3 skin reactions; late toxicity, surveyed after 6 months: late grade 1 and 2 skin toxicity: A: 73.3%, B: 83.3%, *p* = 0.347, ns; late grade 1 and 2 mucosal toxicity: A: 56.7%, B: 73.3%, *p* = 0.405, ns; late grade 2 salivary gland toxicity: A: 13.3%, B: 20%, *p* = 0.488; no grade 3, 4 and 5 skin/mucosal/salivary gland toxicity 4. Lower Hb and leukocyte levels in B, ns; lower urea in A at week 3 (*p* = 0.019), stat. sign
**Mansourian et al. (2015)**	RCT	Head and neck cancer RTx during intervention Included: *n* = 37 Analyzed: *n* = 37 A: *n* = 19 B: *n* = 18 Dropout-rate: overall 0%/ no information	Arm A: Curcuma longa topical gel (dried hydroalcohol derivative of curcuma longa, 0.5% gel) Arm B: Placebo gel Duration: 21 days	T0: Baseline T1-T3: Day 7, 14, 21 1. Mucositis (WHO Mucositis Scale) 2. Burning mouth sensation (VAS) 3. Oral mucosal erythema 4. Oral ulcers	1. Maximum grade of mucositis n (%): grade 1: A: 15 (78.9%), B: 3 (16.7%); grade 2: A: 4 (21.1%), B: 8 (44.4%); grade 3: A: 0 (0%), B: 7 (38.9%); grade 4: A and B: 0**;** frequency of different grades of mucositis: *p* < 0.001; time between treatment initiation and maximum grade of mucositis: *p* = 0.315, ns, time until symptoms started was significantly longer in A (no *p*-value), incidence of maximum intensity of mucositis: T1: A: 4 (21.1%), B: 8 (44.4%); T2: A: 6 (31.6%), B: 4 (22.2%); T3: A: 9 (47.4%), B: 6 (33.3%) 2. Mean (SD): A: 3.7 (2.1), B: 7.9 (2.0), *p* < 0.001 3. Max. size in mm (mean [SD]): A: 4.9 (2.2), B: 8.9 (2.7), *p* < 0.001 4. Max. size in mm (mean [SD]): A: 1.3 (2.7), B: 6.4 (4.2); *p* < 0.001
**Najafizade et al. (2023)**	RCT	Colorectal cancer RCTx during intervention Included: *n* = 44 Analyzed: *n* = 44 A: *n* = 22 B: *n* = 22 Dropout-rate: overall 0%/ no information	Arm A: Curcumin capsules (Curcumin glucuronide, Dineh company) twice a day, 500 mg/d Arm B: Placebo (manufactured by Isfahan School of Pharmacy) Duration: 6 weeks	T0: Baseline T1: week 2 T2: week 4 T3: week 6 T4: week 8 1. Severity of acute radiation enteritis (NIH CTCAE version 4)	1. Intestinal side effects after RTx: blood in stool: A: 1 (4.5%), B: 1 (4.5%), abdominal pain: A: 1 (4.5%), B: 0 (0%), nausea: A: 3 (13%), B: 4 (18%), mucus in stool: A: 0 (0%), B: 2 (9%), diarrhea: A: 2 (9%), B: 1 (4.5%), total: A: 7 (31%), B: 9 (40%), *p* = 0.17, ns; intestinal disorders: grade 1: A: 3 (13%), B: 4 (18%), grade 2: A: 3 (13%), B: 3 (13%), grade 3: A: 1 (4%), B: 1 (4%), grade 4: A: 0 (0%), B: 0 (0%), *p* = 0.17, ns; prevalence of complications in different stages of the disease ns between groups (*p* = 0.321); extent of intestinal involvement in radiation field in cm^3^: A: grade I/II: 221.2, grade III: 465.5, *p* = 0.03, B: grade I/II: 218.1, grade III: 458, *p* = 0.03; frequency of complications over time: most frequent in first two weeks in both groups, persisting until week 3 in B, declining trend in A, ns (no p-value given); all patients cured after eight weeks (*p* = 0.22)
**Nakao and Ueno (2021)**	RCT	Head and neck cancer RTx during intervention (± previous CTx ± surgery) Included: *n* = 27 Analyzed: *n* = 25 A: *n* = 5 B: *n* = 5 C: *n* = 4 D: *n* = 5 E: *n* = 6 Dropout-rate: overall 7.4%, A: 0%, B: 0%, C: 25%, D: 20%, E: 0%	Arm A: Turmeric extract, 160 µg/ml gel Arm B: Chlorhexidine Arm C: Curry leaf Arm D: Propolis Arm E: Placebo Duration: Approx. 1 month	T0: Baseline T1: Assessment of compliance T2: 37.5 ± 11.5d 1. Dryness in the oral cavity (VAS) 2. Pain (VAS) 3. Clearance of oral pathogens after the gel application	1. ns in turmeric group 2. ns in turmeric group, overall statistically sign. decrease with application of moisturizing gel in all participants, *p* ≤ 0.05 3. ns in turmeric group
**Panahi et al. (2014)**	RCT	Solid cancer, mainly colorectal, gastric, and breast cancer CTx during intervention (different CTx, depending on cancer type) ± RTx Included: *n* = 96 Analyzed: *n* = 80 A: *n* = 47 B: *n* = 49 Dropout-rate: overall 16.7%, A: 14.9%, B: 18.4%	Arm A: bioavailability-boosted curcuminoids preparation 180 mg/d, phytosomal preparation of curcuminoids (Meriva®, Indena S.p.A., Italy), curcuminoids complexed with phosphatidylcholine, 300 mg/capsule, overall content of curcuminoids 20%, 3 capsules/day Arm B: Matched placebo Duration: 8 weeks	T0: Baseline T1: Week 2 T2: Week 4 T3: Week 6 T4: Week 8 1. health-related QoL (UW-QoL version 4) 2. serum levels of a panel of mediators implicated in systemic inflammation	1. Sign. increase in QoL in both groups (*p* < 0.001); sign. higher increase in QoL in curcumin group, A: 34.23 (8.23), B: 5.62 (5.02), *p* < 0.001; subgroup analysis: within groups difference ns between patients with and without concomitant RTx (*p* > 0.05) or stratified by cancer type (*p* > 0.05), but sign. in group comparison: colorectal carcinoma: A: 35.00 (5.73), B: 4.62 (5.85), *p* < 0.001; breast cancer: A: 38.80 (3.56), B: 4.67 (4.76), *p* < 0.001; gastric cancer: A: 27.71 (13.07), B: 5.40 (5.77), *p* = 0.005; other: A: 35.44 (6.64), B: 6.31 (4.53), *p* < 0.001; with RTx: A: 35.25 (8.66), B: 3.43 (4.89), *p* < 0.001; without RTx: A: 33.41 (8.63), B: 6.04 (5.22), *p* < 0.001; correlations between QoL and specific serum levels; sign. predictor for QoL by group: TGFβ, IL-8, combined analysis: TGFβ, MCP-1, CGRP 2. Sign. reduction of TNF-α, TGFβ, IL-6, substance P, hs-CRP, CGRP, MCP-1 (*p*'s < 0.005)
**Panahi et al. (2021)a**	RCT	Colorectal cancer CTx during intervention Included: *n* = 72 Analyzed: *n* = 67 A: *n* = 36 B: *n* = 36 Dropout-rate: overall 6.9%, A: 8%, B: 6%	Arm A: Curcuminoids capsules 500 mg/day (C3 Complex®, Sami Labs Ltd., Bangalore, India), also contained piperine (5 mg; Bioperine®, Sami Labs Ltd., Bangalore, India), as a bioavailability enhancer Arm B: Placebo Duration: 8 weeks	T0: Baseline T1: After 8 weeks 1. Biochemical parameters: Erythrocyte sedimentation rate (ESR), serum levels of C-reactive protein (CRP) and 12 pro- and anti-inflammatory cytokines 2. QoL (EORTC-QLQ-C30)	1. Sign. difference in CRP (*p* = 0.002) and ESR (*p* = 0.0001) in T1; IL-1α sign. decreased (*p* = 0.077) 2. Sign. difference in functional scale change (SD): A: -1.42 (3.84), B: -7.95 (3.84), *p* = 0.002; sign. difference in symptom scales (SD): A: -1.06 (6.81), B: -9.62 (6.3), *p* = 0.0001, but both not in every single domain; sign. difference in global quality of life change (SD): A: -4.84 (1.82), B: -6.5 (1.82), *p* = 0.020; sign. difference in overall health during the past week (*p* = 0.021), but not in overall quality of life during the past week (*p* = 0.299); sign. correlation between baseline values and score changes in all QoL scales in A and B: A: *p* < 0.001 in all 3 scales, B: global QoL *p* = 0.002, functional and symptom scale *p* < 0.001 each
**Panahi et al. (2021)b**	RCT	Various cancers CTx during intervention Included: *n* = 80 Analyzed: *n* = 78 A: *n* = 38 B: *n* = 40 Dropout-rate: overall 2.5%, A: 5%, B: 0%	Arm A: Curcumin capsules (500 mg curcuminoids plus 5 mg piperine, Sami Labs, Bangalore, India), 1 capsule every 12 h, 1000 mg/d Arm B: Placebo Duration: 9 weeks	T0: Baseline T1: After 9 weeks T2-T4: Follow-up (clinical symptoms) after 1, 2 and 3 months 1. Health-related QoL (UW-QoL questionnaire, version 3) 2. Hematological and biochemical parameters 3. Clinical symptoms	1. T0: sign. group difference in recovery score: A: 76.32 (23.21), B: 63.13 (24.67), *p* = 0.017, and swallowing score: A: 92.89 (19.85), B: 73.00 (32.44), *p* = 0.002; other items ns (*p*'s > 0.132); T1: sign. group differences in swallowing score: A: 95.00 (14.28), B: 71.25 (37.43), *p* = 0.015, advantage A; quality-of-life score: A: 35.26 (17.67), B: 53.50 (15.28), *p* < 0.001, advantage B; quality-of-life rate score: A: 30.53 (17.85), B: 56.00 (14.46), *p* < 0.001, advantage B; other items ns (*p*'s > 0.065); total QoL score: A: 84.16 (17.04), B: 77.05 (14.66), *p* = 0.083, ns, but sign. improvement in both groups (*p* = 0.036); sign. time effect for pain, activity, recreation, QoL score, QoL rate score, socioemotional function and total QoL (*p*’s < 0.038); sign. time x group interaction for QoL score and QoL rate score (*p*’s < 0.001) 2. Sign. differences in Hb, HCT, LDH, SGOT, ALK 3. Baseline: no sign. group differences in symptoms surveyed (nausea, vomiting, diarrhea, constipation, anorexia, weight loss, itching, insomnia, skin lesion, mouth ulcer, neuropathy, fever, body pain neurological, eye lesion, dry mouth), *p*'s > 0.179; comparison of change in symptoms over time between groups: sign. difference in nausea in 2nd and 3rd month (*p* = 0.013, 0.011), anorexia in 1st, 2nd and 3rd month (*p* = 0.002, 0.009, 0.013), insomnia in 2nd and 3rd month (*p* = 0.046, 0.005), mouth ulcers in 1st and 2nd month (*p* = 0.008, 0. 005), neuropathy in the 1st, 2nd and 3rd (*p* = 0.018, 0.001, 0.014), body pain in the 2nd and 3rd (*p* = 0.031, 0.008), neurological symptoms in the 1st and 3rd month (0.021, 0.001) and dry mouth in the 3rd month (*p* = 0.024) in curcumin group
**Passildas-Jahanmohan et al. (2021)**	RCT	Prostate cancer CTx during intervention (± previous surgery/RTx/ hormonotherapy) Included: *n* = 50 Analyzed: *n* = 44 (mITT ) A: *n* = 26 B: *n* = 24 Dropout-rate: overall 12% (unknown group allocation)	Arm A: Docetaxel + Curcumin 500 mg capsules, 6 g/day, 4 capsules three times a day Arm B: Docetaxel + Placebo Duration: For 7 consecutive days (from day − 4 to day + 2 with CTx at day 0) every 3 weeks, 6 cycles of CTx	T0: Baseline T1-T2: PSA measured every 3 weeks, Tumor evaluation cycle 3 and 6 Follow-up: every 3 months until progression 1. Time to progression, reported as progression-free survival 2. Compliance 3. Overall survival 4. PSA change 5. Safety 6. Curcumin absorption 7. Quality of life (QLQ-C30 and QLQ-PR25)	Difference on main objective considered statistically sign. at *p* < 0.03, standard *p* value used for other secondary objectives, results from interim analysis did not justify continuing the study 1. Median PFS in months: A: 3.7, B: 5.3, *p* = 0.75, ns; PFS after 6 months: A: 31.8%, B: 45.5%, *p* = 0.35, ns; 38.6% of patients were alive without any documented progression 2. A: 96.38%, B: 95.55%, *p* = 0.68 3. Median OS in months: A: 15.8, B: 19.8, *p* = 0.50, ns; 4. After 12 months: A: 60.1%, B: 80.0%, *p* = 0.17; after 24 months: A: 20.0%, B: 29.3%, *p* = 0.62 patients with PSA decline of ≥ 50%: A: 6, B: 12; no p-value given; PSA evolution over entire treatment period ns (*p* = 0.88) 5. Less lymphopenia (*p* = 0.023) and less hypocalcemia (*p* = 0.021) in A, statistically sign. difference; incidence of grade 3– 4 AEs: ns, no SAEs attributed to curcumin 6. Nonhydrolyzed form not detectable (< 2 ng/ml) in either arm; T0: no hydrolyzed curcumin detected; cycle 1, 3 and 6: sign. difference (*p* = 0.002, 0.03, 0.03), in B hydrolyzed curcumin value always under limit of quantification; no cumulative effect of curcumin between cycles 1– 3 and cycle 3– 6 (*p* = 0.26 and *p* = 0.99), but interindividual variability (*p* = 0.0065) 7. QLQ-C30: *p* = 0.49, QLQ-PR25: *p* = 0.47, ns
**Patil et al. (2015)**	RCT	Head and neck cancer RCTx during intervention Included: *n* = 20 Analyzed: *n* = 20 A: *n* = 10 B: *n* = 10 Dropout-rate: overall 0%/ no information	Arm A: Freshly prepared curcumin mouthrinse 0.004%, to be used in 1:5 dilution for 1 min, three times daily Arm B: Commercially available chlorhexidine mouthwash 0.2% to be used for 1 min, in 1:1 dilution, thrice daily Duration: 20 days	T0: Baseline T1: Day 10 T2: Day 20 1. Mucositis (WHO Scale, OMAS) 2. Pain (NRS)	Comparison baseline – T2: 1. Erythema score decrease: A: 0.50, B: 0.70, *p* = 0.050, according to authors statistically sign.; ulceration score decrease: A: 0.4, B: 0.9, *p* < 0.001; WHO score decrease: A: 2.0, B: 2.6, *p* = 0.003, statistically sign 2. NRS decrease: A: 2.65, B: 0.95, *p* < 0.001, statistically sign
**Ramezani et al. (2023)**	RCT	Head and neck cancer RTx during intervention Included: *n* = 45 Analyzed: *n* = 37 A: *n* = 15 B: *n* = 15 C: *n* = 15 Dropout-rate: overall 17.8%, A: 13.3%, B: 20% C: 20%	Arm A: Curcumin mouth rinse 0.1%, made from 100 mg curcumin powder (Karen, Iran), freshly prepared daily, 3 × daily 10 ml to be used for 1 min Arm B: Sinacurcumin soft gel capsules 40 mg per day, curcuminoids as nanomicelles (SinaCurcumin®) Arm C: Placebo mouth rinse Duration: 21 days	T0: Baseline T1: Week 1 T2: Week 2 T3: Week 3 1. Mucositis (WHO Oral Toxicity Scale) 2. Pain (NRS)	1. T1 and T2: group differences ns; T3: according to authors A and B sign. better mean value than C (*p* < 0.001) at week 3; free from ulcerations: A: > 33%, B: > 15%, C: 0%; score reduction T1—T3 compared to baseline (T0-T1, T0-T2, T0-T3, T1-T2, T1-T3, T2-T3): within-group: A: *p*'s < 0.018, B: T0-T1: *p* = 0.20, from T0-T2: *p*'s < 0.027, C: *p*'s > 0.051; group comparison: T0-T1: *p* = 0.981, T0-T2: *p* = 0.150, T0-T3: *p* = 0.002, T1-T2: *p* = 0.074, T1-T3: *p* < 0.001, T2-T3: *p* = 0.029, according to authors sign. reduction in A and B compared to C 2. T1 and T2: group differences ns; T3: according to authors A and B sign. better mean value than C at week 3 (*p* < 0.001); score reduction from T1—T3 compared to baseline (T0-T1, T0-T2, T0-T3, T1-T2, T1-T3, T2-T3): within-group: A: *p*'s < 0.036, B: *p*'s < 0.008, C: T0-T1: *p* = 0.29; T2-T3: *p* = 0.81, others: *p*'s < 0.019; group comparison: *p*'s < 0.01; according to authors sign. reduction in A and B compared to C
**Rao et al. (2014)**	RCT	Head and neck cancer RTx ± CTx during intervention (± previous surgery) Included: *n* = 80 Analyzed: *n* = 79 A: *n* = 39 B: *n* = 40 Dropout-rate: overall 1.3%, A: 2.5%, B: 0%	Arm A: One turmeric capsule (400 mg) dissolved in approx. 80 ml of boiled and cooled water, mouth swished 4 times with 10 ml for about 2 min, 6 times a day Arm B: Povidone-iodine solution diluted 1:100 (betadine 1 ml and 100 ml water), 10 ml twice a day Duration: 6 weeks	T0: Baseline T1-T7: Weekly, for 7 weeks 1. Oral mucositis (RTOG guidelines) 2. Toxicity	1. T1-7: A < B, *p* < 0.001, taken from graph only, no values specified; intolerable mucositis: A: 14/39, B: 34/40, *p* < 0.0001 2. Treatment interruption/delay necessary: A: 7 (17.95), B: 9 (24), ns; treatment days lost: mean (SD): A: 7(0), B: 7.25 (0.56), ns; weight loss: mean (SD): A: 3.92 (2.13), B: 4.45 (2.15), *p* < 0.001, statistically sign
**Ryan et al. (2013)**	RCT	Breast cancer RTx during intervention Included: *n* = 35 Analyzed: *n* = 30 A: *n* = 14 B: *n* = 16 Dropout-rate: overall 14.3%, A: 17.6%, B: 11.1%	Arm A: Curcumin C3 Complex® oral 500 mg capsules (≥ 95% curcuminoids), 4 capsules 3 times daily (6 g/d) Arm B: Placebo Duration: 4 – 7 weeks	T0: Baseline From T1: Weekly after every fifth RTx session, At end of RTx, Post-RTx: 1 month and 6 months post-RTx 1. Radiation dermatitis severity (RDS scoring scale) 2. Moist desquamation 3. Redness measurement 4. Pain (c 5. Toxicity	1. Difference in mean RDS A–B (SD; 95% CI): -0.8 (0.8, -1.4; -0.2); *p* = 0.008; arm*week interaction: A vs B (without consideration of time): *p* = 0.963, but sign. arm*week interaction, *p* = 0.007 2. Percentage of patients: A: 28.6%, B: 87.5%, *p* = 0.002 3. A vs B (without consideration of time): *p* = 0.328, arm*week interaction: *p* = 0.588 4. Difference in mean pain baseline – end-RTx (95% CI): pain at RTx site: A: 1.929 (0.855, 3.002), B: 1.313 (0.365, 2.260); *p* = 0.504; other pain: A: –0.286 (-1.309, 0.738), B: –0.563 (-1.432, 0.307), *p* = 0.967; MPQ-SF: MPQ total: A: 4.643 (2.045, 7.241), B: 2.875 (1.543, 4.207), *p* = 0.144; perceived pain: A: 0.857 (0.358, 1.356), B: 0.813 (0.523, 1.102), *p* = 0.644; sensory pain: A: 3.286 (1.217, 5.354), B:1.750 (0.827, 2.673), *p* = 0.081; affective pain: A: 0.500 (0.061, 0.939), B: 0.375 (-0.008, 0.758), *p* = 0.550, ns; types of pain: gnawing, aching, splitting (A > B, *p* < 0.021), otherwise ns 5. ns (nausea, vomiting, upset/depressed, shortness of breath, memory, lack of appetite, diarrhea, change in urination, skin problems in treatment area, disturbed sleep, fatigue, general activity, mood, work, relationships, walking and quality of life)
**Ryan Wolf et al. (2017)**	RCT	Breast cancer RTx during intervention Included: *n* = 686 Analyzed: *n* = 686 (ITT, PP: 578) A: *n* = 283 B: *n* = 295 Dropout-rate: overall 15.7%, A: 17.7%, B: 13.7%	Arm A: Curcumin C3 Complex® capsules (450 mg curcumin, 40 mg Dimethoxycurcumin, 10 mg Bisdemethoxycurcumin), 4 capsules 3 times daily (6 g/d) Arm B: Placebo Duration: During RTx + 1 week	T0: Baseline From T1: Weekly after every fifth RTx session, At end of RTx, Post-RTx: 1-week post-RTx 1. Radiation dermatitis severity (RDS scoring scale) 2. Moist desquamation 3. Pain at RTx site (MPQ-SF) 1 week post-RTx 4. Skin-related Quality of Life (Skindex-29) 1 week post-RTx 5. Severity of AEs	1. Most common locations for worst radiation dermatitis axillary region (B 44.2%; A 40.4%), inframammary fold (B 44.6%, A 42.6%), ns in mean RDS score end-RTx: regression coefficient B (95%CI): 0.044 (-0.101, 0.188), *p* = 0.552; trend towards less RDS > 3.0 in A (A 7.4%; B 12.9%; *p* = 0.082), ns; post-RTx RDS score: ns (*p* = 0.489); sign. effects due to irradiated area and RTx scheme (*p* < 0.001) 2. 63 patients with moist desquamation, inframammary fold most common location (B 49.2%; A 42.8%); ns in presence of moist desquamation end-RTx (B 9.54%; A 12.2%; *p* = 0.324); post-RTx also ns (B 16.7%; A 14.8%; *p* = 0.565); no correlation between RDS > 3.5 and moist desquamation (*p* = 0.062); 23.3% of patience with moist desquamation had RDS > 3.5; 69.6% had RDS 2.0; 2.5 or 3.0 3. ns: sensory *p* = 0.714, affective *p* = 0.068, perceived *p* = 0.481, total *p* = 0.255, influence of RTx regime on affective pain: increased with Canadian fractionation, but decreased with conventional fractionation over time 4. ns: emotion *p* = 0.286, symptom *p* = 0.665, composite *p* = 0.407, worry *p* = 0.863 5. ns
**Ryan Wolf et al. (2020)**	RCT	Breast cancer RTx during intervention (± prior CTx) Included: *n* = 188 Analyzed: *n* = 169 (ITT: multiple imputation) A: *n* = 64 B: *n* = 63 Arm C: *n* = 61 Dropout-rate: overall 10.1%, A: 7.8%, B: 7.9% C: 14.8%	Arm A: Psoria-Gold® curcumin gel (4% curcumin) Arm B: HPR Plus™ (medical device recommended for atopic dermatitis and radiation dermatitis) Arm C: Placebo Duration: During RTx + 1 week	T0. Baseline From T1: From week 3 weekly after every fifth RTx session, at end of RTx, Post-RTx: 1 week and 2 weeks post-RTx 1. Radiation dermatitis severity (RDS score, NIH CTCAE, self-reported skin symptoms) 2. Pain at RTx site (self-report Pain Diary and Skin-Pain Inventory) 3. Moist desquamation 4. Time to implementation of standard care 5. Acceptability and blinding (feedback questionnaire) 6. Correlation of patient-reported measures Subgroup analysis: breast field separation ≥ 25 cm (*n* = 22 patients)	1. RDS score EndRTx mean (95% CI): A: 2.68 (2.49, 2.86), B: 2.64 (2.45, 2.82), C: 2.63 (2.44, 2.83), *p* = 0.929, ns; RDS peak in week 6, ns; NIH CTCAE mean grade 2.2, 77% patients having grade 1 or 2 radiation dermatitis 2. ns in pain diary scores and self-reported pain scores; Pain Diary score EndRTx mean (95% CI): A: 1.96 (1.31, 2.62), B: 2.28 (1.63, 2.92), C: 2.44 (1.74, 3.14), *p* = 0.840; skin severity EndRTx (Skin-Pain Inventory) mean (95% CI): A: 1.93 (1.63, 2.23), B: 1.76 (1.47, 2.06), C: 2.08 (1.77, 2.39), *p* = 0.139; self-reported skin problems in Skin-Pain Inventory: ns, peak in week 5; in A and B more patients with none or very mild itchiness (73% and 76%) or redness (37% and 39%) than in C (51%, *p* = 0.009 and 19%, *p* = 0.044); pain severity EndRTx (Skin-Pain Inventory) mean (95% CI): A: 1.47 (1.12, 1.81), B: 1.44 (1.10, 1.78), C: 1.64 (1.28, 2.00), *p* = 0.685; highly significant arm by time interaction for mean pain diary scores (*p* < 0.001) in longitudinal analyses for medium pain, A lower than B and C in week 6 3. Moist desquamation EndRTx in % (n): A: 25.42% (15), B: 20.34% (12), C: 22.64% (12), *p* = 0.805, ns; sign. difference in NCORP sites (36%, 17%, 8%, 21%, 26%, *p* = 0.047) 4. Standard care initiated in 57%: A: 63%, B: 53%, C: 54%, ns in time to implementation; “standard care” regimens varied by NCORP site, sixteen different topical modalities, most commonly prescribed in all arms: aquaphor (20–32%), silver sulfadiazine cream (26–27%), topical steroid (8–12%) 5. Despite color difference only 30% correctly chose assigned study arm (A: 45%, B: 25%, C: 18%); presumed curcumin: B: 39%, C: 67%; > 50% would use their assigned topical agent in the future 6. Self-reported skin problems and pain showed stronger correlation with each other than with RDS scores at week 4 and week 5 during RTx; strong association of self-reported skin problems and pain at week 4 (*p* = 0.697, *p* < 0.0001) and week 5(*p* = 0.766, *p* < 0.0001); RDS scores at week 4 and week 5 weakly correlated with self-reported skin problems (*p* = 0.510 and *p* = 0.454, *p* < 0.0001) and pain scores (*p* = 0.368 and *p* = 0.347, *p* < 0.0001) Subgroup analysis (*n* = 22): increased breast field separation positively correlated with increased radiation dermatitis severity (*p* = 0.018); ANOVA showed significant differences in RDS scores (*p* = 0.022) and pain scores (*p* = 0.046) at EndRTx; A: lower RDS scores over time than B and C (*p* = 0.024); A and B had lower pain scores over time than C (*p* = 0.036)
**Saadipoor et al. (2018)**	RCT	Prostate cancer RTx during intervention Included: *n* = 64 Analyzed: *n* = 64 (ITT, PP: 63) A: *n* = 33 B: *n* = 31 Dropout-rate: overall 1.6%, A: 3%, B: 0%	Arm A: Nanocurcumin capsules (SinaCurcumin®, 100% curcumin) 120 mg/d Arm B: Placebo Duration: Starting 3 days before and during RTx	T0: Baseline From T1: Weekly during RTx, Once after RTx (26 fractions) 1. Radiation-induced proctitis (CTCAE) 2. Radiation-induced cystitis (CTCAE) 3. Hematological parameters (measured before, 2 × weekly during and once after RTx) 4. Treatment response based on diffusion-weighted MRI (as percent of ADC increase approx. 3 months after RTx, in IMRT patients only, PP evaluation)	1. ≥ grade 1: A: 45.5%, B: 58.1%, *p* = 0.313; ≥ grade 2: A: 15.2%, B: 9.7%, *p* = 0.709; duration: mean number of weeks (SD): A: 1.2 (1.5), B: 1.3 (1.4), *p* = 0.651, ns 2. ≥ grade 1: A: 87.9%, B: 74.2%, *p* = 0.161; ≥ grade 2: A: 42.4%, B: 25.8%, *p* = 0.162; duration: mean number of weeks (SD): A: 3.3 (1.6), B: 2.5 (2), *p* = 0.309, ns 3. ns 4. sign. increase in ADC values of lesions in both groups after RTx; mean ADC increase (95%CI): B: 228 (63, 393), *p* = 0.011; A: 269 (149, 389), *p* < 0.001; ns between pre- and post-RT ADC values of contralateral normal tissue (*p* = 0.390); ns in ADC value increase between groups (*p* = 0.574); mean percentage ADC increase: A: 33.8%, B: 48.4%; mean group difference: 14.6% (95% CI: -52.4, 23.1)
**Saghatelyan et al. (2020)**	RCT	Breast cancer CTx during intervention Included: *n* = 150 Analyzed: *n* = 150 (PP: 127) A: *n* = 62 B: *n* = 65 Dropout-rate: overall 11.3%, A: 13.3%, B: 9.3%	Arm A: Curcumin + Paclitaxel 300 mg injection solution of Curcumin© dissolved in 250 ml physiological saline (95% curcuminoids), once every 7 d Arm B: Paclitaxel + “placebo” (riboflavin solution for injection, 200 mg in 20 ml) Duration: 12 weeks	T0: Pretest and inclusion in study, T1: After 1 week (randomization, QoL questionnaire, first drug administration), T2-T11: Weekly drug administration with laboratory control + AE recording, T12: Final examination after 12 weeks, T13 and T14: Follow-up after 16 and 24 weeks 1. Objective response rate at 4 and 12 weeks after last administration of each intervention (ORR, defined as CR or PR confirmed 4 weeks after first response) 2. Progression-free survival (PFS) 3. Time to tumor progression (TTP) 4. Safety and tolerance of therapy (by CTCAE, time to treatment failure TTF) 5. Karnofsky performance status 6. ECOG performance status 7. Patient-reported quality of life (QLQ und QLQ-C30)	1. ORR at 16 weeks: ITT ORR, overall (95% CI): 42% (34.0 – 50.0); stable disease: 29.3% (A: 24.0%, B: 34.7%, *p* = 0.9257); progressive disease: 12.7% (A: 6.7%, B: 18.7%, *p* = 0.9877); complete response 0%; ORR sign. higher in A than in B, and sign. higher than 1 (2.6, *p* = 0.0081); A: 50.7%, B: 33.3%, *p* = 0.0145; PP ORR: A: 61.3%, B: 38.5%, *p* = 0.0041, stat. sign.; ORR at 24 weeks: ITT ORR: A: 29.0%, B: 20.0%, *p* = 0.0911, ns; stable disease: A: 12, B: 15; progressive disease: A: 15, B: 24; complete response: A: 1, B: 0; PP ORR: A: 44.9%, B: 27.8%, *p* = 0.0337; ORR sign. higher in A than in B; RECIST score at 4 weeks follow-up significantly different (*p* = 0.0085) compared to baseline in both groups; RECIST score after 3 months follow-up in A significantly higher (*p* < 0.001) than baseline, in B significantly lower (*p* < 0.05) than baseline, but ns; difference between groups at this time point (*p* = 0.076), RECIST score negative in B, positive in A; ECOG: ns in ORR in patients with ECOG score 0–1 at baseline, with ECOG score 2 higher ORR in A (*n* = 8, 72.3%) than in B (*n* = 1, 6.3%; *p* < 0.001); small subgroups (11 and 16 patients, respectively); ns in ORR with respect to other clinical characteristics at baseline or previous treatments (with few patients in each subgroup) 2. After 24 weeks: 60% PFS, PFS median (95% CI) in weeks: overall: 25.6 (23.0 – 28.2), A: 27, B: 24.6, *p* = 0.3495, HR 1.278, ns 3. Median TTP in weeks: A: 27, B: 24.6, *p* = 0.3026, HR: 1.319, ns 4. ns in treatment completion and duration, ns in TTF (*p* = 0.4556), patients with delay in treatment due to thrombocytopenia (3), rash (1), pneumonia (1), patient request (1); ns between groups in treatment-emergent adverse events (TEAEs); fatigue: A: 4.0%, B: 13.3%, *p* = 0.0548, ns 5. Stat. sign. difference, interaction time & intervention, presumed advantage for A (*p* = 0.046) 6. ns (*p* = 0.9244) 7. ns in overall QoL (*p* = 0.49), stat. sign. difference in physical conditions, advantage A (*p* = 0.028)
**Sandoughdaran et al. (2021)**	RCT	Bladder cancer CTx during intervention Included: *n* = 26 Analyzed: *n* = 26 (PP: 23) A: *n* = 12 B: *n* = 14 Dropout-rate: overall 11.5%, A: 16.7%, B: 7.1%	Arm A: Nanocurcumin capsules 80 mg (SinaCurcumin®), twice daily Arm B: Placebo (same manufacturer) Duration: Gemcitabine i.v. on day 1 and 8, every 21d; Creatinine clearance > 60 ml/min: Cisplatin, < 60 ml/min: Carboplatin on day 1 and 2 every 21 days	T0: Baseline T1: 4 weeks after end of treatment (CTx + intervention) 1. Tumor response (complete clinical response to CTx, cystoscopy by blinded, independent urologist) 4 weeks after end of treatment 2. CTx-induced nephrotoxicity (CTCAE) 3. Hematologic toxicity (CTCAE)	1. Tumor downstaging to pT0: A: 50%, B: 30.8%, *p* = 0.417, ns; ns between patients with cisplatin and carboplatin (*p* = 0.999) 2. Nephrotoxicity grade 3/ 4, n (%): A: 2 (16.7%), B: 1 (7.1%), *p* = 0.580, ns 3. Hematologic toxicity grade 3/ 4, n (%): leukopenia: A: 1 (8.3%), B: 2 (14.3%), *p* = 0.999, ns; neutropenia: A: 7 (58.4%), B: 5 (35.7%), *p* = 0.249, ns; anemia: A: 1 (8.3%), B: 1 (7.1%), *p* = 0.999, ns; no cases of thrombocytopenia in both groups
**Santosa et al. (2022)**	RCT	Multiple myeloma CTx during intervention Included: *n* = 33 Analyzed: *n* = 23 A: *n* = 17 B: *n* = 16 Dropout-rate: overall 30.3%, A: 29.4%, B: 31.3%	Arm A: Melphalan 4 mg/m^2^ and prednisone 40 mg/m^2^ for 7 days (single dose) and curcumin 1000 mg BCM95, Biocurcem® 8 g/d (4 capsules, twice daily) Arm B: Melphalan, prednisone and placebo Duration: 28d each for 4 cycles, 112 days total	T0: Baseline T1: Day 28 T2: Day 56 T3: Day 84 T4: Day 112 (every 28d for 4 cycles) 1. Overall remission, stable disease 2. Laboratory data: NF-κB, VEGF, TNF-α, IL-6, LDH, CRP	1. OR: A: 75%, B: 33.3%, *p* = 0.01, statistically sign.; stable disease: A: 25%, B: 66.7%, *p* = 0.01, stat. sign 2. Sign. decrease in NF-κB, VEGF, TNF-α; sign. decrease in IL-6 levels in subgroup analysis; TNF-α level had sign. correlation with remission, OR = 1.35, (95%CI: 1.03–1.76), *p* = 0.03
**Shah et al. (2020)**	RCT	Head and neck cancer RTx during intervention Included: *n* = 74 Analyzed: *n* = 68 (mITT, PP: 17) A: *n* = 37 B: *n* = 37 Dropout-rate: overall 8.1%	Arm A: Curcumin (0.1%) mouth rinse freshly made from curcumin nanoparticles powder, sodium benzoate and clove oil, 10 ml thrice daily Arm B: Benzydamine (benzydamine hydrochloride BP 0.15%) mouth rinse solution, commercially available (COOLORA™) Duration: 7 days	T0: Baseline T1-T6: After 1 week each 1. RIOM scores (Radiation-induced oral mucositis), especially effect on onset, progression, and severity of RIOM (WHO criteria)	1. PP median (IQR): baseline: A: 0 (0), B: 0 (0), *p* = 1.00; T1: A: 0 (0–0.75), B: 0 (0), *p* = 0.71; T2: A: 0.50 (0–1.75), B: 1.00 (1.00–1.00), *p* = 0.39; T3: A: 1.00 (0.25–1.75), B: 0.40 (1.00–2.5), *p* = 0.40; T4: A: 1.00 (0.25–2), B: 2.00 (1.00–2.5), *p* = 0.26; T5: A: 1.00 (0.25–1.0), B: 1.00 (1.00–2.5), *p* = 0.06; T6: A: 1.00 (0.25–1.75), B: 1.00 (1.00–2.5), *p* = 0.14; ns between groups; PP: incidence RIOM: A: 75%, B: 100%, *p* = 0.206; course: in both groups sign. increase in WHO scores; comparison in groups at single time points, by post hoc analyses – results not presented in an understandable way, according to authors ns, also ns between groups at single time points; severity, highest scores: ≥ 3: A: 0, B: 2, *p* = 0.15, ns; ≤ 2: A: 8, B: 7, no *p*-value given; ME: median onset in days: A: 21.00, B: 7.00, *p* = 0.001; HR for onset: 0.5, *p* = 0.039, *p* = 0.001 – Risk for onset 50% lower in A than in B; for further endpoints no analysis by mITT possible due to missing data
**Soni et al. (2021)**	RCT	Oral cancer RCTx during intervention Included: *n* = 42 Analyzed: *n* = 42 A: *n* = 21 B: *n* = 21 Dropout-rate: overall 0%/ no information	Arm A: 500 mg BTF capsules (BCM-95®), turmeric extract with 95% curcuminoids and essential oil, produced by extraction of dried C. longa rhizomes 2 × daily p.o. (1 g/d) Arm B: 500 mg BTF 3 × daily (1,5 g/d) Arm C: Placebo Duration: 6 weeks	T0: Baseline T1-T6: Weekly 1. Incidence and severity of CTx-induced oral mucositis (WHO Mucositis Scale) 2. Dysphagia 3. Oral pain (NRS) 4. Dermatitis 5. Significant weight loss (> 3 kg from baseline) 6. Compliance with RTx (> 3 consecutive missed RTx appointments)	1. At baseline, all grade 0 with progression to grade 1 after 2 weeks of CTx + RTx from week 4, decrease in incidence and severity in A and B compared to C, in B sign. improvement compared to C (p = 0.017); mucositis grade 3 after 5 weeks: A: 20%, B: 15%, C: 35%, no statistical values given for this; after 6 weeks: grade 2: A: 75%, B: 80%, C: 35%, grade 3: A: 25%, B: 20%, C: 65%; comparison of severity levels: A and B: *p* = 0.705, ns, A and C: *p* = 0.011, B and C: *p* = 0.004, stat. sign 2. After 6 weeks: grade 2: A: 75%, B: 80%, C: 40%; grade 3: A: 25%, B: 20%, C: 60%; comparison: A and B: *p* = 0.705, ns, A and C: *p* = 0.025, B and C: *p* = 0.010, stat. sign 3. Week 1: all grade 0; after 2 weeks progression to grade 1; from week 3 decrease in A and B compared to C; week 4: grade 3: A: 5%, B: 0%, C: 15%, no statistical values given for this; week 6: grade 2: A: 65%, B: 70%, C: 30%; grade 3: A: 35%, B: 30%, C: 70%; comparison of severity levels: A and B: *p* = 0.736, not sign., A and C: *p* = 0.027, B and C: *p* = 0.011, stat. sign 4. Week 5: grade 2: A: 80% (*p* = 0.035), B: 75% (*p* = 0.017), C: 100%, stat. sign.; week 6: grade 2: A: 90%, B: 95%, C: 70%; grade 3: A: 10%, B: 5%, C: 30%; comparison: A and B: *p* = 0.548, A and C: *p* = 0.114, ns, B and C: *p* = 0.037, stat. sign 5. Gastric tube needed: A: 25%, B: 20%, C: 60%; comparison: A and B: *p* = 0.705, A and C: *p* = 0.025, B and C: *p* = 0.010, stat. sign.; hospitalization during RTx: A: 20%, B: 15%, C: 55%; comparison: A and B: *p* = 0.677, A and C: *p* = 0.022, B and C: *p* = 0.008, stat. sign.; weight loss in kg mean (SD): A: 3.18 (1.3), B: 3.03 (1.35), C: 4.55 (1.78); comparison: A and B: *p* = 0.946, A and C: *p* = 0.014, B and C: *p* = 0.006, stat. sign.; weight loss ≥ 3 kg: A: 60%, B: 25%, C: 75%,; comparison: A and B: *p* = 0.311, A and C: *p* = 0.025, B and C: *p* = 0.002, stat. sign 6. Treatment completed without interruptions: A: 80%, B: 85%, C: 45%; comparison: A and B: *p* = 0.677, A and C: *p* = 0.022, B and C: *p* = 0.008, stat. sign
**Talakesh et al. (2022)**	RCT	Breast cancer RTx during intervention Included: *n* = 60 Analyzed: *n* = 60 A: *n* = 20 B: *n* = 20 C: *n* = 20 Dropout-rate: overall 0%/ no information	Arm A: RTx + 80 mg/d nanocurcumin capsules twice daily (ExirNanoSina Company and Jaber Ebne Hayyan Pharmaceutical Company) Arm B: RTx + placebo (from soybean oil, same manufacturer as B) + All: Aloe vera gel Duration: from the start of treatment until 2 weeks after RTx	T0: Baseline T1-T7: Weekly 1. Efficacy of curcumin nanomicells for reduction of radiation-induced skin reactions (RISR) (per RTOG skin toxicity) 2. Pain level (Likert 0 to 10 or Behavioral Pain Scale)	1. T1: grade 0: A: 100%, B: 100%, *p* = 1.000; T2: grade 0: A: 76.19%, B: 71.42%, grade 1: A: 23.80%, B: 28.57%, *p* = 0.726; T3: grade 0, A: 52.38%, B: 47.61%, grade 1: A: 47.61%, B: 52.38%, *p* = 0.758; T4: grade 0: A: 38.09%, B: 38.09%, grade 1: A: 61.90%, B: 52.38%, grade 2: A: 0%, B: 9.52%, *p* = 0.658; T5: grade 0, A: 4.76%, B: 0%, grade 1: A: 66.66%, B: 66.66%, grade 2, A: 28.57%, B: 33.33%, *p* = 1.000; T6: grade 0: A: 9.52%, B: 0%, grade 1: A: 38.09%, B: 33.33%, grade 2: A: 52.38%, B: 66.66%, *p* = 0.461; T7: grade 0: A: 9.52%, B: 0%, grade 1: A: 47.61%, B: 14.28%, grade 2: A: 42.85%, B: 85.71%, *p* = 0.011; sign 2. Pain level median (P10, P90): T1: A: 0 (0, 1.6), B: 0 (0, 2.8), *p* = 0.147, ns; T2: A: 0 (0, 3.8), B: 1 (0, 4.6), *p* = 0.031, sign.; T3: A: 0 (0, 3.8), B: 2 (0, 5), *p* = 0.045, sign.; T4: A: 1 (0, 4), B: 3 (0.2, 4.8), *p* = 0.010, sign.; T5: A: 2 (0, 4.8), B: 4 (1, 5), *p* = 0.019, sign.; T6: A: 1 (0, 4), B: 4 (0, 5), *p* = 0.009, sign.; T7: A: 1 (0, 5), B: 4 (1.2, 5), *p* = 0.002, sign
**Thambamroong et al. (2022)**	RCT	Head and neck cancer CTx and/or RTx during intervention Included: *n* = 20 Analyzed: *n* = 20 (ITT) A: *n* = 10 B: *n* = 10 Dropout-rate: overall 10% A: 10%, B: 10%	Arm A: Curcumin 500 mg capsules twice daily 4 capsules via stomach tube, 4000 mg/day Arm B: Placebo capsules (from probiotics) Duration: 8 weeks	T0: Baseline T1: Week 4 T2: Week 8 (for BIA, laboratory values every two weeks) 1. Body composition (lean body mass or muscle mass, body fat mass, and basal metabolic rate [BMR]) 2. Handgrip muscle strength 3. BMI 4. Absolute lymphocyte count level 5. Safety and toxicity	1. Mean change from baseline and percent change (95%CI): T2: muscle mass change in kg: A: 0.46 (-0.2, 1.12), B: -1.05 (-2.34, 0.24), *p* = 0.03; muscle mass change in %: A: 2.16 (-0.75, 5.07), B: -3.82 (-8.2, 0.57), *p* = 0.019, sign.; body fat mass change in kg: A: -0.39 (-1.16, 0.38), B: 0.98 (-2.08, 0.12), *p* = 0.334; body fat mass change in %: A: -0.51 (-21.89, 20.86), B: -8.97 (-19.43, 1.49), *p* = 0.432, ns; BMR, change in kcal: A: 5.8 (-23.49, 35.09), B: -22.2 (-56.59, 12.19), *p* = 0.178; BMR, change in %: A: 0.54 (-1.6, 2.67), B: -1.61 (-4.05, 0.84), *p* = 0.153; ns 2. T2: handgrip muscle strength in kg: A: 0.61 (-2.17, 3.39), B: -0.62 (-3.03, 1.79), *p* = 0.956; handgrip muscle strength in %: A: 2.73 (-9.62, 15.07), B: -0.82 (-10.16, 8.52), *p* = 0.935; ns 3. T2: BMI change in kg/m^2^: A: -0.1 (-0.71, 0.51), B: -0.88 (-1.85, 0.08), *p* = 0.206; BMI change in %: A: -0.63 (-4.1, 2.84), B: -4.2 (-8.64, 0.25), *p* = 0.344; ns 4. T2: cell count change in cells/mm^3^: A: -227.7 (-694.7, 239.3), B: -888.9 (-1439.45, -338.35), *p* = 0.053, ns; cell count change in %: A: -4.55 (-45.78, 36.69), B: -48.31 (-68.62, -28), *p* = 0.05; sign. (according to the authors) 5. AEs: nausea A: 40%, B: 20%, *p* = 0.628; diarrhea: A: 0%, B: 30%, *p* = 0.211; headache: A: 20% B: 10%, *p* = 1.000; all AEs mild (grade 1); no serious AEs (hepatitis, acute kidney injury); ns
**Thomas et al. (2023)**	RCT	Head and neck cancer RTx during intervention (± concurrent CTx) Included: *n* = 92 Analyzed: *n* = 88 A: *n* = 46 B: *n* = 46 Dropout-rate to week 6: overall 4.3%, A: 2.2%, B: 6.5%, Week 7: overall 52.2%, A: 54.3%, B: 50.0%	Arm A: Turmeric powder in 400 mg capsules (Himalaya Drug Company), dissolved in 80 ml boiled and cooled water, rinse mouth with 10 ml for 2 min, repeat 4x, 6 × daily (1 h before, 1, 2, 4, 6 h after RTx, 1 × before sleep) Arm B: Benzydamine mouth rinse 10 ml each with the same amount of water, rinse once, 6xdaily (as in A) Duration: 6 to 7 weeks	T0: Baseline From T1: Weekly until end of RTx 1. Oral health (Oral Health Assessment Tool – OHAT) 2. Mucositis (WHO oral toxicity criteria) 3. Oral dysfunction (using Patient Reported Oral Mucositis Symptom – PROMS Scale and Xerostomia Short Form Inventory) 4. Weight loss in kg 5. Number of days with treatment interruption of RTx	1. Increase in score from baseline to week 7: A: from 2.33 (1.49) to 9.29 (2.61), B: from 2.82 (1.18) to 11.32 (1.78), sign. difference; between A and B, ANOVA over time, *p* = 0.001 2. Incidence grade 3 mucositis: A: 4.34% (T4), 8.7% (T6), B: 4.35% (T2), 89.1% (T4); grade 4: A: 0% (total), B: 2.2% (T3), 30.4% (final RTx); mean onset of tolerable mucositis in weeks (grades 1 and 2): A: 3.44 ± 1.2, B: 1.21 ± 0.49, *p* = 0.001; intolerable mucositis (grades 3 and 4): A: 5.92 ± 1.32, B: 3.76 ± 0.84, *p* = .001), sign. differences; End-RTx mean mucositis score: A: 1.90 ± 0.70, B: 3.64 ± 0.49, *p* = .001, sign. difference; mean score: increase in score from baseline to week 7: A: from 0 (0) to 1.9 (0.7), B: from 0 (0) to 3.64 (0.49), *p* = 0.001 3. Sign. lower mean score in all weeks of measurement in A (*p* = 0.001) 4. Weight loss in kg from baseline to week 7: A: from 58.62 (10.73) to 56.52 (10.06), B: from 58.95 (8.60) to 54 (8.74), *p* = 0.001 5. Days with treatment interruption: A: 0.47 (1.40), B: 3 (3.04), *p* = 0.002

ADC: apparent diffusion coefficient, ADT: androgen deprivation therapy, AEs: Adverse events, BCR: biochemical recurrence, BIA: bioelectrical impedance analysis, BMR: basal metabolic rate, BTF: bio-enhanced turmeric formulation, CR: complete response, CTx: chemotherapy, EBRT: External Beam Radiation Therapy, HR: hazard ratio, IAD: intermittent androgen deprivation, ITT: intention-to-treat, mITT: modified intention-to-treat, MPQ-SF: McGill Pain Questionnaire Short-form, NCI CTCAE; National Cancer Institute Common Terminology Criteria for Adverse Events, NCI-CTC v.2: National Cancer Institute Common Toxicity Criteria version 2, NRS: Numeric Rating Scale, ns: not significant, OMAS: Oral Mucositis Assessment Scale, OR: objective response, ORR: objective response rate, OS: overall survival, pCR: pathological complete response, PFS: progression-free survival, PP: per protocol, PR: partial response, PSA: prostate specific antigen, QoL: quality of life, RCT: Randomized Controlled Trial, RCTx: radiochemotherapy, RDS: radiation dermatitis severity, RIOM: Radiation-induced oral mucositis, RTOG: Radiation Therapy Oncology Group, RTx: radiotherapy, SD: standard deviation, SEM: standard error of the mean, SI: Symptom Inventory, TTP: time to tumor progression, VAS: visual analog scale

## Results

**Fig. 1:**
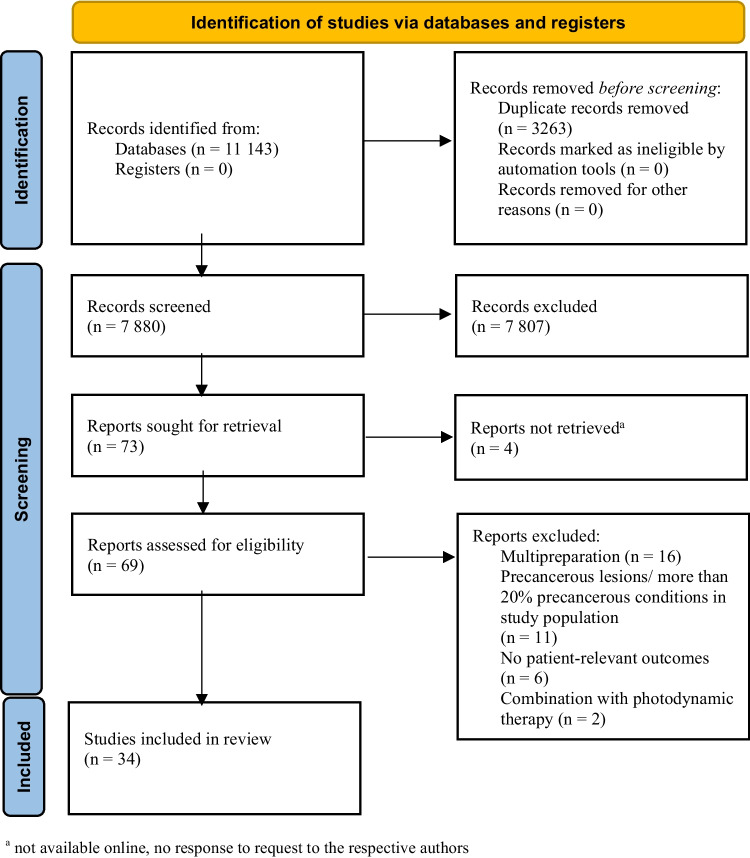
Consort diagram (PRISMA 2020**) **[66]

The systematic research revealed 11,143 results. At first, duplicates were removed leaving 7880 studies. After screening title and abstract, 69 studies remained to complete review (Fig. 1). Finally, 34 publications were included in this review.

### Characteristics of included studies

In all relevant studies, 2580 patients were included, of which 2449 were analyzed due to 322 dropouts. The age of the included patients ranged from 22 to 90 years with a range of age within the studies from 25 to 63 years. 1489 participants were female, 963 were male, and 128 had no gender listed. For detailed characteristics of the studies and respective results, see Table 3 and 4.

**Table 4:** Overview of the results and respective methodological quality

Outcome	Reference	In favor of curcumin	No effect	Overall RoB
Oral symptoms	Arun et al	**X**		High
	Charantimath et al	**X**		High
	Delavarian et al	**X**		High
	Fardad et al	**X**		High
	Kia et al	**X**		Moderate
	Mansourian et al	**X**		Moderate
	Patil et al	**X**		High
	Ramezani et al	**X**		High
	Rao et al	**X**		High
	Soni et al	**X**		Moderate
	Thomas et al	**X**		High
	Shah et al.^a^	**X**	**X**	High
	Nakao et al		**X**	High
Pain associated with oral symptoms	Charantimath et al	**X**		High
	Fardad et al	**X**		High
	Patil et al	**X**		High
	Ramezani et al	**X**		High
	Soni et al	**X**		Moderate
	Kia et al.^a, b^	**X**	**X**	Moderate
	Nakao et al		**X**	High
Skin symptoms	Talakesh et al	**X**		Moderate
	Ryan et al.^a^	**X**	**X**	High
	Ryan Wolf (2020) et al.^a^	**X**	**X**	High
	Ryan Wolf (2017) et al		**X**	Moderate
Pain associated with skin symptoms	Talakesh et al	**X**		Moderate
	Ryan et al	**X**		High
	Ryan Wolf (2020) et al		**X**	High
	Ryan Wolf (2017) et al		**X**	Moderate
Weight loss	Delavarian et al	**X**		High
	Rao et al	**X**		High
	Soni et al	**X**		Moderate
	Thomas et al	**X**		High
	Chaiworramukkul et al		**X**	High
Body composition	Thambamroong et al.^a^	**X**	**X**	Moderate
	Chaiworramukkul et al		**X**	High
OS	Howells et al	**X**		High
	Gunther et al		**X**	Moderate
	Passildas-Jahanmohan et al		**X**	Moderate
PFS	Gunther et al		**X**	Moderate
	Howells et al		**X**	High
	Passildas-Jahanmohan et al		**X**	Moderate
	Saghatelyan et al		**X**	Moderate
Disease progression with PSA	Choi et al.^a, b^	**X**	**X**	High
	Hejazi (2016) et al		**X**	High
	Passildas-Jahanmohan et al		**X**	Moderate
Tumor response	Howells et al	**X**		High
	Santosa et al	**X**		High
	Saghatelyan et al. ^a, b^	**X**	**X**	Moderate
	Choi et al		**X**	High
	Gunther et al		**X**	Moderate
	Saadipoor et al		**X**	Moderate
	Sandoughdaran et al		**X**	High
	Kumar et al.^d^	**-**	**-**	High
QoL and ADL	Panahi (2014) et al	**X**		High
	Hejazi (2013) et al.^a^	**X**	**X**	High
	Panahi (2021)a et al.^b^	**X**	**X**	High
	Panahi (2021)b et al.^a^	**X**^c^	**X**	Moderate
	Saghatelyan et al.^a^	**X**	**X**	Moderate
	Choi et al		**X**	High
	Howells et al		**X**	High
	Passildas-Jahanmohan et al		**X**	Moderate
	Ryan Wolf (2017) et al		**X**	Moderate

^a^ outcome measured with different instruments/ different symptoms/ different subgroups, not all of which showed significant results; ^b^not significant at all measured time points; ^c^also in favor of comparison in 2 of the measured scores; ^d^no p-value reported

### Excluded studies

A total of 35 RCTs were excluded according to the predefined eligibility criteria. Among them, 11 had less than 80% cancer patients, 6 did not report patient-relevant outcomes, and 2 used curcumin as a photosensitizer to support photodynamic therapy without evaluating the effect of curcumin separately. Sixteen RCTs were excluded due to multiple interventions, as individual parts of the intervention could not be analyzed separately. Excluded studies are listed in table S2 (online resource 2).

### Risk of bias in included studies

**Fig. 2:**
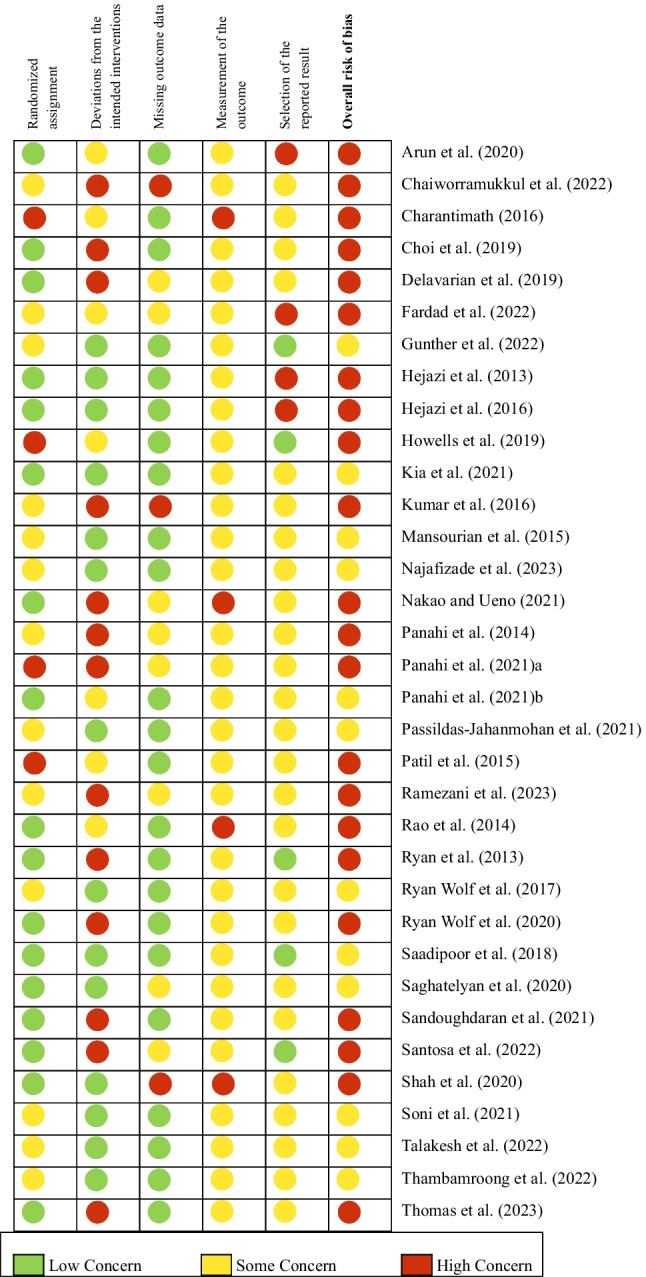
RoB Assessment of RCTs

The methodological quality was assessed using Cochrane revised Risk of Bias Tool 2.0. The results are presented in Fig. 2 and table S1 (online resource 1). Twelve of the included studies had a moderate and 22 had a poor quality. Besides small sample sizes and/or no power analyses [10–32], the major methodological issues are missing double blinding [11, 17, 18, 25, 26, 29, 33–36] or possibility of unblinding (e.g. different forms of application or intake frequencies between groups; [13, 26, 34, 37]), unreliable concealment method [36], no ITT analyses [10, 12, 13, 18, 21–23, 26, 27, 29, 33, 34, 36, 38, 39], low report quality [11, 12, 15–22, 25, 34], and moderate or high drop-out [10, 12, 14, 17, 18, 22, 26–29, 32, 35, 36, 38–40]. Randomization and comparability of baseline values were often questionable with little information given [10–13, 15–17, 19–22, 24, 25, 27, 34, 37–39]. Due to curcumins typical, bright yellow color, it remains unclear if blinding was ensured in all forms of application, as only 2 studies evaluated blinding [27, 35]. Six studies declared a conflict of interest [14, 23, 31, 39–41], 9 had the medication sponsored by the manufacturer [15, 16, 22, 29, 33, 36, 37, 41, 42]. Other specific drawbacks can be found in the supplement (table S1, online resource 1).

### Efficacy of curcumin therapy

#### Specific symptoms, associated pain, weight alteration and body composition

##### Oral symptoms and associated pain:

Mucositis and other oral symptoms were assessed in 13 RCTs using different scales [11–13, 19, 21, 25, 26, 30, 33, 34, 36, 37, 43]. Only one showed no significant group differences [21].

Seven RCTs utilized the WHO Oral Mucositis Scale [11, 19, 25, 30, 33, 37, 43]. All of them demonstrated a significant advantage of curcumin. Two showed a benefit over chlorhexidine after 20 days (*p* = 0.003; [25]) and after 2 weeks (*p* < 0.001; [11]), one over placebo after 3 and 4 weeks and at 2 months (*p*’s < 0.001; [33]). Another RCT found an advantage after 6 weeks between a lower (1 g/d) and higher (1.5 g/d) dosage curcumin group and placebo group (*p*’s < 0.017; [37]). There was an advantage of nanocurcumin compared to placebo after 1, 4 and 7 weeks (*p*’s < 0.022). Subgroup analysis also showed this among patients treated solely with chemotherapy (CTx) (*p*’s < 0.008) and patients receiving both radiotherapy (RTx) and CTx (*p*’s < 0.012; [43]) at some of the investigated times. Mansourian et al. [19] found a significant group difference in the frequency of maximum mucositis grades (*p* < 0.001) and a delay in symptom onset comparing curcumin and placebo gel (*p* < 0.050). The time between treatment initiation and highest grade of mucositis did not differ significantly (*p* = 0.315). Shah et al. [30] found a significant difference in median symptom onset (21 vs. 7 days, *p* = 0.001) and significant hazard ratio (HR) for onset (*p*’s < 0.039), indicating a lower risk of onset in curcumin group compared to benzydamine. No difference was found in median WHO score up to 6 weeks, WHO scores ≥ 3, and incidence of oral mucositis (*p*’s > 0.060) in the per protocol (PP) population (*n* = 17).

Three RCTs utilized the WHO Oral Toxicity Scale [13, 26, 36]. One showed a significant benefit of curcumin over chlorhexidine on day 3 (*p* = 0.050; [13]), another one over benzydamine in mean onset of tolerable and intolerable mucositis, mean mucositis score at the end of RTx, and increase in mean score until week 7 (*p*’s = 0.001; [36]). Ramezani et al. found a significant score reduction after 3 weeks in both curcumin groups (mouth rinse, nanocurcumin capsules) compared to placebo (*p* < 0.001; [26]).

Four RCTs assessed erythema and ulcerations [11, 13, 19, 25], all found curcumin advantageous. Three used the Oral Mucositis Assessment Scale [11, 13, 25] and discovered a significant difference in both erythema and ulcerations after 2 weeks compared to placebo (*p*'s < 0.001; [11]) and after 20 days compared to chlorhexidine (*p*’s < 0.050; [25]), in erythema on day 3 and in ulceration on day 5 compared to Mucosamin® and chlorhexidine (*p*’s < 0.040; [13]). Mansourian et al. [19] found a significant difference between curcumin and placebo gel in mean maximum size for erythema and ulcerations (*p*’s < 0.001) after 3 weeks.

Three RCTs utilized National Cancer Institute Common Terminology Criteria for Adverse Events (NCI CTCAE), National Cancer Institute Common Toxicity Criteria version 2 scale (NCI-CTC v.2), or Radiation Therapy Oncology Group (RTOG) guidelines [12, 33, 34]. Two demonstrated a significant benefit of curcumin [33] or nanocurcumin [12] compared to placebo after 21 days, 28 days and 2 months (*p*’s < 0.001; NCI CTCAE subjective scale; [33]) and weekly up to 6 weeks (*p* < 0.050; NCI-CTC v.2; [12]). The third trial found less intolerable mucositis in curcumin mouthwash compared to povidone-iodine in weekly assessments up to 7 weeks (*p* < 0.001; RTOG guidelines; [34]).

Four RCTs assessed other oral symptoms [19, 21, 36, 37]. Three of them found an advantage in curcumin [19, 36, 37]. A significant difference in dysphagia and radiation-induced dermatitis was observed among oral cancer patients between a lower (1 g/d) and higher curcumin dosage (1.5 g/d) and a placebo group (*p*’s < 0.037; [37]). Thomas et al. [36] found a significant difference between curcumin and benzydamine group in Oral Health Assessment Tool score increase from baseline to week 7 (*p* < 0.050), ANOVA over time (*p* = 0.001), PROMS Scale and Xerostomia Short Form Inventory throughout the study period (*p* = 0.001). Mansourian et al. [19] evaluated burning mouth sensation using visual analog scale (VAS) and found that curcumin had a significant advantage over placebo gel (*p* < 0.001) after 3 weeks. Only Nakao and Ueno [21] assessed clearance of oral pathogens after gel application and oral dryness after approximately 1 month using the VAS. However, they found no significant difference in the turmeric gel group (*p* > 0.050) compared to chlorhexidine, curry leaf, propolis, and placebo group.

Out of 11 RCTs evaluating pain [11, 13, 21, 25–27, 31, 35, 37, 40, 43], 7 focused on pain associated with mucositis from RTx or CTx in head and neck cancer patients [11, 13, 21, 25, 26, 37, 43]. Six used the numeric rating scale (NRS) for assessment and found a significant difference [11, 13, 25, 26, 37, 43], one used VAS and found no significant group difference [21]. Curcumin was found to be more advantageous than chlorhexidine gluconate after 2 weeks (*p* < 0.001; [11]), at day 3 compared to Mucosamin® and chlorhexidine (*p* = 0.020; [13]), and after 20 days compared to chlorhexidine mouthwash (*p* < 0.001; [25]). Soni et al. [37] observed an advantage of low (1 g/d) and high dosage curcumin (1.5 g/d) compared to placebo group (*p*’s < 0.027) after 6 weeks. Ramezani et al. reported a significant score reduction of both curcumin mouthwash and nanocurcumin capsule groups compared to placebo after 3 weeks (*p* < 0.001; [26]). Similarly, Kia et al. [43] found that nanocurcumin was superior to placebo at week 7 (*p* = 0.001) and in a subgroup analysis of patients who received CTx after 2, 4, and 7 weeks (*p*’s < 0.005). However, there was no significant difference in patients who underwent both CTx and RTx (*p* > 0.050). There was no significant difference in pain evaluated by the VAS (*p* > 0.050) in Nakao and Ueno. Nonetheless, the use of moisturizing gel resulted in an overall significant decrease in pain in all participants (*p* ≤ 0.05; [21]).

##### Skin symptoms and associated pain:

Radiation dermatitis severity (RDS) and associated pain were evaluated in 4 RCTs [27, 31, 35, 40] with only one study finding significant group differences in both RDS and pain scale [31]. One study partly found significant group differences for RDS [27] and another one for associated pain [35], one found no significant difference [40].

Nanocurcumin﻿ was superior to placebo in week 7 in RTOG skin toxicity score (*p* = 0.011) and from the second to seventh week in pain level median (P10, P90; *p* < 0.045; Behavioral Pain Scale; [31]). Ryan et al. found an advantage of curcumin compared to placebo in mean RDS at the end of treatment (*p* = 0.008) and fewer incidence of moist desquamation (*p* = 0.002), but not in redness (*p*’s > 0.328) and pain measurements (*p*’s > 0.081; [27]). Ryan Wolf et al. (2020) [35] reported significantly more patients with none or very mild itchiness or redness in both curcumin and HPR Plus™ ("FDA-approved medical device recommended for atopic dermatitis and radiation dermatitis” [35]) groups compared to placebo (*p*’s < 0.044), but no significant difference in RDS score (*p* = 0.929) or moist desquamation (*p* = 0.805). There was a highly significant arm by time interaction for mean pain diary scores (*p* < 0.001) with lower scores in curcumin than in HPR Plus™ and placebo group in week 6.

No significant group difference was observed between curcumin and placebo in mean RDS scores and presence of moist desquamation at the end and after RTx (*p*’s > 0.324), and in the MPQ-SF 1 week after RTx in sensory, affective, perceived, and total pain (*p* > 0.068; [40]).

##### Weight alteration and body composition:

Weight alteration was evaluated in 5 RCTs [10, 12, 34, 36, 37], 4 showing significantly less weight loss in the curcumin group [12, 34, 36, 37]. An advantage was found after 7 weeks compared to povidone-iodine (*p* < 0.001; [34]) and benzydamine (*p* = 0.001; [36]), after 6 weeks in nanocurcumin compared to placebo (*p* = 0.003; [12]), and in lower (1 g/d) and higher (1.5 g/d) curcumin dosage compared to placebo (*p*’s < 0.014; [37]). Soni et al. [37] found the same results for weight loss higher than 3 kg (*p* = 0.311 vs. *p* = 0.025 vs. *p* = 0.002), need of a gastric tube (*p* = 0.705 vs. *p* = 0.025 vs. *p* = 0.010), and hospitalization during RTx (*p* = 0.677 vs. *p* = 0.022 vs. *p* = 0.008). Chaiworramukkul et al. [10] found no significant difference between curcumin and placebo group after 8 weeks in weight loss (*p* = 0.810), change of body fat mass, skeletal muscle mass, BMI, hand grip muscle strength, and basal metabolic rate (*p*’s > 0.119). Thambamroong et al. [32] found a significant difference between curcumin and placebo group after 8 weeks in mean change in muscle mass (*p*’s < 0.030), but not in body fat mass, basal metabolic rate change, handgrip muscle strength, and BMI change (*p*’s > 0. 153).

#### Survival, disease progression, and tumor response

##### Overall survival (OS) and progression-free survival (PFS):

OS and PFS or time to tumor progression (TTP) were analyzed in 4 RCTs [14, 17, 24, 41] involving patients with either colorectal, rectal, prostate or breast cancer. Three found no significant group difference between curcumin and placebo in five-year OS and five-year PFS (*p*’s > 0.70; [14]), in median OS, OS at 12 or 24 months, median PFS and PFS after 6 months (*p*’s > 0.17; [24]), and in median PFS and mean TTP using curcumin intravenous and riboflavin as placebo (*p*’s > 0.30; [41]). Howells et al. [17] found no significant difference in HR for PFS (Intention-to-treat [ITT]: *n* = 27; HR: 0.571; *p* = 0.200; PP: *n* = 24; HR: 0.549; *p* = 0.183), but for OS in both ITT (*n* = 27; HR: 0.339; *p* = 0.016) and PP population (*n* = 24; HR: 0.271; *p* = 0.004) in favor of curcumin group compared to control.

##### Disease progression with PSA:

Three RCTs assessed PSA change from baseline or progression rate comparing a curcumin and a placebo group [15, 24, 38], with only 1 finding a significant difference [38]. Choi et al. found a significantly lower PSA progression in the curcumin group (10.3% vs. 30.2%, *p* = 0.026) after 6 months. No significant difference was found in PSA change from baseline (*p*’s > 0.054) and in subgroup analyses (*p*’s > 0.856; [38]), neither did Hejazi et al. after 3 months (*p* = 0.780; [15]) or Passildas-Jahanmohan et al. after 6 cycles of CTx (*p* = 0.880; [24]).

##### Tumor response:

Objective response rate (ORR), overall response (OR), stable disease (SD), and time to treatment failure (TTF) were analyzed in 3 RCTs [17, 29, 41], all showing significant advantages for curcumin. They found an advantage compared to placebo in OR (75% vs.33.3%, *p* = 0.010) and SD (25% vs. 66.7%, *p* = 0.010) within 112 days [29], compared to control in ORR at cycle 12 of CTx (53.3% vs. 11.1%, *p* = 0.039; [17]), and compared to a riboflavin group in ORR (ITT: *n* = 150, 50.7% vs. 33.3%, *p* = 0.015; PP: *n* = 127, 61.3% vs. 38.5%, *p* = 0.004) at 16 weeks, but neither in rates of SD (24.0% vs. 34.7%, *p* = 0.926) or TTF (*p* = 0.456; [41]).

Choi et al. found no significant difference for curcumin compared to placebo in off-treatment duration in patients receiving intermittent androgen deprivation (*p* = 0.482; [38]).

Tumor and nodal response, general treatment response, and pathological complete response (pCR) were analyzed by 4 RCTs [14, 18, 28, 42]. Three of them did not find a significant difference [14, 28, 42], 1 did not report *p*-values [18]. Studies showed no difference between a nanocurcumin and a placebo group in increase of apparent diffusion coefficient values in diffusion-weighted MRI three months after RTx (*p* = 0.574; [42]) and tumor downstaging to pT0 in cystoscopy 4 weeks after end of treatment (50% vs. 30.8%, *p* = 0.417; [28]). There was no significant difference in pCR rates at the time of surgery, pathological tumor stage, tumor regression grade, and five-year cumulative incidence of local regional and distant failure comparing curcumin and placebo (*p*’s > 0.180; [14]). Kumar et al. detected no evidence of disease after one year in 66.7% of the curcumin group and in 56.7% of the control group using the WHO response criteria but did not report *p*-values [18].

#### Quality of life and activities of daily living (ADL)

The effects of curcumin treatment on QoL were assessed by 9 RCTs utilizing various questionnaires [16, 17, 22–24, 38–41]. One found a significantly higher increase in QoL in curcumin group compared to placebo after 8 weeks (*p* < 0.001) and in several subgroup analyses (*p*’s < 0.001; UW-QoL version 4; [22]). Four RCTs partly found an advantage of curcumin compared to placebo [16, 23, 39, 41]. There was an advantage 3 months after RTx in urinary (*p* = 0.011), but not in bladder or treatment-related symptoms, nor in sexual activity (*p*’s > 0.155; EORTC QLQ-PR25; [16]). Studies also reported an advantage after 8 weeks in various QoL scales including functional scale change, symptom scales, global QoL change, and overall health (*p*’s < 0.021), but not in overall QoL over the past week (*p* = 0.299; QLQ-C30; [39]). Curcumin was superior compared to riboflavin as placebo after 12 weeks in physical conditions (*p* = 0.028) and Karnofsky performance status (time x intervention; *p* = 0.046), but not in overall QoL (*p* = 0.490; QLQ-C30) and ECOG performance status (*p* = 0.924; [41]). Panahi et al. (2021)b used the UW-QoL questionnaire, version 3, and found a significant positive effect for curcumin after 9 weeks on swallowing score (*p* = 0.015), but also a significant negative effect in QoL score and QoL rate score (p’s < 0.001). Other items showed no significant group differences (*p*'s > 0.065), but there was a significant time effect for pain, activity, recreation, QoL score, QoL rate score, socioemotional function, and total QoL (*p*’s < 0.038), and a significant time x group interaction for QoL score and QoL rate score (*p*’s < 0.001; [23]).

Four studies did not find a significant difference [17, 24, 38, 40] between a curcumin and placebo or control group in health-related QoL after 6 months in FACT-P, IPSS total, IPSS QoL, and IIEF-15 (*p*’s > 0.386; [38]), in EORTC QLQ-PR25 or QLQ-C30 (*p*’s > 0.470) after 6 cycles of RTx [24], in QLQ-C30 after 24 weeks (*p* = 0.248, [17]), and in the Skindex-29 questionnaire for skin-related QoL 1 week after RTx (*p*’s > 0.286; [40]).

#### Side effects and treatment interruptions

##### Side effects related to curcumin:

Besides significantly more frequent vomiting compared to CTx only (*p* = 0.037) in Kumar et al. [18], one study reported several side effects “possibly or probably related to curcumin” [17] in patients treated with FOLFOX, bevacizumab, and curcumin. Most of them were gastrointestinal, but without significant difference between curcumin and control group. The most common was diarrhea, followed by nausea, dyspepsia, oral mucositis, constipation, vomiting, anorexia, abdominal pain, acute kidney injury, bloating, dry mouth, and flatulence [17]. Another study observed mild gastrointestinal side effects in 20% of the patients in curcumin group with no dropout due to adverse events (AEs), but did not analyze safety as a specified endpoint and did not report *p*-values [22].

##### Side effects related to tumor therapy other than oral or skin symptoms:

Four RCTs evaluated safety, clinical symptoms or acute and late RTx toxicity and found significant group differences during the trial [18, 23, 24, 38], mostly indicating fewer side effects of concomitant CTx, RTx or radiochemotherapy in the curcumin group.

Significantly fewer AEs (*p* = 0.0349; [38]), lower rates of lymphopenia and hypocalcemia (*p*’s < 0.023; [24]), and a significant symptom decrease for nausea, anorexia, insomnia, mouth ulcers, neuropathy, body pain, neurological symptoms, and dry mouth (*p*’s < 0.046; [23]) were reported in curcumin compared to placebo. One study found significantly less grade 1 blood urea toxicity (*p* = 0.019), but more vomiting in curcumin compared to CTx only [18].

##### Treatment interruptions:

Two studies found significantly less treatment interruptions in a curcumin mouth rinse group compared to a benzydamine group (*p* = 0.002; [36]) and in a lower (1 g/d) and a higher (1.5 g/d) curcumin dosage group compared to placebo (*p*’s < 0.022; [37]).

## Discussion

The findings of the included studies indicate that the administration of curcumin may offer a potential benefit with respect to oral symptoms and weight loss. Regarding other patient-relevant endpoints, the results were inconclusive or demonstrated no significant difference in comparison to the control group. The included studies showed considerable heterogeneity, with notable discrepancies in their study designs, rendering a direct comparison challenging. It is also conceivable that not all the included studies provided reliable results due to the considerable variation in methodological quality (see table S1, online resource 1 for details). These two factors may have contributed to inconsistent results. Looking at thematically similar recent SRs, we found that the inclusion criteria appeared to be less broad in terms of symptom-related outcomes, pain, tumor progression, survival, and quality of life [44], which were our priorities, and included more studies of laboratory parameters or tumor markers [45]. Others were limited to a specific cancer type [46] or further narrowed the endpoints, particularly to oral mucositis [47–49]. Furthermore, a considerable number of new publications on curcumin in cancer therapy have emerged in recent years. In view of this, we decided to conduct this SR with the inclusion and exclusion criteria we defined accordingly (Table 1). A comprehensive presentation of the results and the respective methodological quality is provided in Table 4. The disparate outcomes of the included studies, in conjunction with the methodological quality, as well as considerations regarding sufficient dosage and the associated risks, will be discussed in the following paragraphs.

The impact of curcumin on oral symptoms induced by tumor therapy was assessed by 13 RCTs [9–21], with 7 evaluating the effect on associated pain [10, 11, 13, 14, 16, 20, 21]. All but one [20], which was characterized by a small sample size, found an advantage of curcumin. Similarly, other SRs and meta-analyses that restricted their investigation to the use of curcumin for oral symptoms yielded comparable outcomes [47–49]. However, the included studies show a mostly high [9, 10, 13, 15–21] or moderate [11, 12, 14] risk of bias.

The same applies to studies that have investigated weight loss [10, 12, 34, 36, 37]. Here too, most results show an advantage for curcumin with less weight loss in the curcumin group [12, 34, 36, 37] and a moderate [37] or high [10, 12, 34, 36] risk of bias. Only one found no effect on both weight loss and other body composition parameters [10]. Two SRs and meta-analyses on the impact of curcumin on oral mucositis also concluded that curcumin has a beneficial effect on weight loss [47, 48].

Given the limited number of studies that have investigated body composition [10, 32] with moderate [32] or high [10] risk of bias, it is not possible to make any definitive statements in this regard.

The results for the remaining endpoints were also inconclusive, with no discernible advantage for curcumin. RTx-associated skin symptoms and associated pain in breast cancer patients were evaluated by 4 RCTs [27, 31, 35, 40]. The studies show a moderate [31, 40] or high [27, 35] risk of bias. The two larger studies found no group differences, but both had high dropout and used PP analysis for most of the included endpoints [35, 40].

Ten RCTs analyzed whether curcumin could have an impact on survival or mitigate disease progression in cancer patients [14, 15, 17, 18, 24, 28, 29, 38, 41, 42]. Three studies evaluated disease progression using PSA in prostate cancer patients [15, 24, 38] with only one showing significantly lower progression in the curcumin group [38]. The studies show a high [15, 38] and moderate [24] risk of bias. Four RCTs [14, 17, 24, 41] evaluated OS or PFS in different cancer entities. One study showed a significant effect of curcumin on OS in metastatic colorectal cancer patients [17], but 2 found no significant difference on this endpoint in locally advanced rectal cancer or in metastatic castration-resistant prostate cancer patients [14, 24]. None of the studies found a significant group difference in PFS [14, 17, 24, 41]. Included studies show a moderate [14, 24, 41] or high [17] risk of bias. Seven RCTs evaluated tumor response [14, 17, 18, 28, 29, 41, 42], 3 found advantages for curcumin in ORR, OR, SD, and TTF [17, 29, 41].

The effects of curcumin treatment on QoL were assessed by 9 RCTs utilizing various questionnaires [16, 17, 22–24, 38–41], 4 of them did not find a significant group difference [17, 24, 38, 40].

Overall, 6 studies found no significant effect of curcumin in all or almost all investigated endpoints [10, 14, 20, 24, 40, 42], two of them terminated early due to lack of significant results [14, 24].

Curcumin has been used in various application forms. Studies with topical applications all found an advantage of curcumin [11, 13, 19, 21, 25, 26, 30, 34–36]. Only one RCT utilized intravenous application, which also discovered a benefit of curcumin on the examined endpoints [41]. In oral administration, dosages varied from 500 [20, 39] to 8000 mg per day [14, 29] for curcumin, and from 40 [26] to 180 mg per day [22] for modified curcumin. Soni et al. compared 1000 or 1500 mg curcumin per day and placebo and found both curcumin doses to be superior to placebo in most endpoints [37]. In the studies finding no effects [10, 14, 20, 24, 40, 42], dosages from 500 [20] to 8000 mg [14] or 120 mg nanocurcumin per day [42] were used.

Overall, it remains uncertain whether the duration of application, dosages, and curcumin levels attained in the blood were sufficient in all included studies to demonstrate any effect of curcumin administration. Curcumin absorption also seems to vary significantly among individuals and is challenging to predict [24].

Due to its low bioavailability, high doses seem necessary when using pure curcumin for it to be detected in the plasma. Conversely, higher doses, particularly when combined with additives or modifications for enhanced bioavailability, must be evaluated in terms of safety. The included studies showed that AEs were mostly gastrointestinal [17, 18, 22], including vomiting in one study [18]. Further AEs have been reported in publications with lower evidence level [50–59] or preclinical trials [60], as mentioned in the current guideline on complementary medicine in the treatment of oncology patients [8]. The European Food Safety Authority (EFSA) recommends an Acceptable Daily Intake (ADI) of 3 mg/kg body weight per day for curcumin as a food additive (E100) for permanent intake. It does not apply to curcumin with improved bioavailability due to the presumably higher plasma levels [61]. The German Federal Institute for Risk Assessment has urged additional research on curcumin intake, especially when combined with piperine, to assess potential hepatotoxicity and toxicity thresholds. [62].

Considering that curcumin is a potent antioxidant [2], there is evidence that nutritional supplements containing antioxidants can reduce oxidative damage from CTx and RTx, but also reduce the efficacy of RTx [63]. In vitro or in vivo studies indicate an interaction between curcumin and various chemotherapeutic agents, with curcumin reducing the effect, depending on dosage and time of curcumin administration [8]. Two studies demonstrated that the use of antioxidant supplements before or during CTx or RTx is associated with an increased hazard of recurrence and death [64], a higher total mortality, and lower recurrence-free survival in breast cancer patients [65]. Therefore, antioxidant supplementation during CTx or RTx should be closely monitored [65].

### Limitations of this work

This review has some limitations to be considered. Only studies on adult cancer patients published in German and English until January 2024 and designed as RCTs were included. Studies investigating precancerous lesions, primary prevention, or the use of multi-preparations were excluded from the review. Similarly, studies utilizing alternative surrogate markers for tumor progression other than PSA were not included, in accordance with the regulation of the German S3 guideline on Complementary Oncology [8] which also accepts only PSA and none of the other tumor markers due to its high acceptance as a marker in prevention, surveillance, and during treatment.

Furthermore, the validity of this review is limited due to small participant groups and subgroups, as well as varying characteristics such as cancer types, interventions, scales, and different types of curcumin administration and dosages. Due to the heterogeneity of the studies, resulting in a poor comparability, a meta-analysis was not conducted. Instead, the studies were summarized as a SR.

## Conclusion

Although studies showed some beneficial effects of curcumin administration on cancer therapy-related side effects, especially oral symptoms and weight loss, there were clear methodological weaknesses decreasing the reliability of these results. Furthermore, the studies included in this analysis had heterogeneous designs, evaluated different scores when assessing the same endpoints, and many were of poor quality. Various standard therapies, including different chemotherapeutic agents and RTx regimens, were utilized, which makes it difficult to compare.

More extensive research with higher methodological quality is necessary to give conclusive recommendation on the use of curcumin. Future studies may benefit from the use of larger sample sizes in accordance with a prior power analysis, consequent double blinding and reliable concealment methods, proper randomization, and ITT analysis for statistical analyses. Furthermore, it would be prudent to restrict the scope of individual studies to specific types of cancer and specific parallel standard therapies. In addition, determining appropriate dosages, dosage forms, and durations of use for curcumin is essential to ensure the possibility of an effect, although the potential risks of higher dosages must be considered. Direct comparisons between the use of plain curcumin and curcumin with increased bioavailability would be beneficial to more accurately assess the effects and risks associated with modified curcumin.

Apart from mild gastrointestinal symptoms, the included studies do not report side effects resulting from the administration of curcumin. However, serious AEs associated with the use of curcumin have been reported in other publications. Due to its antioxidant properties, curcumin may potentially interfere with the efficacy of CTx or RTx.

In view of the unclear efficacy and possible risks, the clinical use of curcumin in cancer therapy should be critically scrutinized according to the current state of knowledge.

## Supplementary Information

Below is the link to the electronic supplementary material.

Supplementary file1 (PDF 300 KB)

Supplementary file2 (PDF 189 KB)

## Data Availability

No datasets were generated or analysed during the current study.
