# Supplementary material for: Curcumin as a complementary treatment in oncological therapy: a systematic review
Source: Eur J Clin Pharmacol. 2024 Oct 19;81(1):1–33. doi: 10.1007/s00228-024-03764-9 (PMC11695395; doi:10.1007/s00228-024-03764-9)
Supplement: Supplementary file 1 — Supplementary file1 (PDF 300 KB) [file 228_2024_3764_MOESM1_ESM.pdf]

Supplementary Information 1

Curcumin as a Complementary Treatment in Oncological Therapy: A Systematic Review

Supportive Care in Cancer

Lisa C. Gutsche, Jennifer Dörfler, MSc., Prof. Dr. med. Jutta Hübner

Corresponding Author: Lisa C. Gutsche, Klinik für Innere Medizin II, Hämatologie und Internistische Onkologie, Universitätsklinikum Jena, Am Klinikum 1, 07747 Jena, Germany, lisacgutsche@gmail.com

Table S1: RoB Assessment on included studies

Reference	Study type	Standardized rating of risk of bias	Additional comments on methodology
Arun et al. (2020)	RCT	RoB<br>Randomised assignment: low<br>Deviations from the intended interventions: some concerns<br>Missing outcome data: low<br>Measurement of the outcome: some concerns<br>Selection of the reported result: high<br><br>Overall risk of bias: high	PRO:<br>Methodological quality: ethics vote, power analysis, 4x4 block randomization by independent statistician<br><br>Compliance: high compliance/acceptable dropout, dropout rate 4.7% (therapy declined)<br><br>CONTRA:<br>Methodological quality: no double blinding (only patients blinded), no ITT, no statistical comparison between demographic variables - apparent differences in cancer treatment and cancer type, as well as stage of cancer and also alcohol/smoking/pan chewing, very little baseline information provided<br><br>Statistical analysis: very simplified statistics, extremely similar results for subjective and objective scale, one p-value for all comparisons of the gradings<br><br>Report quality: no information on baseline value of mucositis (presumably 0%, but not clearly communicated), unclear timing of randomization, dropout time unclear for randomization and group membership
Chaiworramukkul et al. (2022)	RCT	RoB<br>Randomised assignment: some concerns<br>Deviations from the intended interventions: high<br>Missing outcome data: high<br>Measurement of the outcome: some concerns	PRO:<br>Methodological quality: ethics vote, baseline characteristics comparable (no sign. differences, but small sample, apparently quite different), double blinding<br><br>CONTRA:

		Selection of the reported result: some concerns<br><br>Overall risk of bias: high	<u>Sample:</u> small sample size, no power analysis<br><br><u>Compliance:</u> high dropout (6 loss to 2nd follow-up, 1 drug intolerance, 1 no clinical benefit)<br><br><u>Statistical analysis:</u> no ITT<br><br><u>Report quality:</u> little information on blinding
Charantimath (2016)	RCT	RoB<br>Randomised assignment: high<br>Deviations from the intended interventions: some concerns<br>Missing outcome data: low<br>Measurement of the outcome: high<br>Selection of the reported result: some concerns<br><br>Overall risk of bias: high	PRO:<br><u>Methodological quality:</u> ethics vote, active control<br><br><u>Dropout/Compliance:</u> no dropout (or no information on dropout)<br><br>CONTRA:<br><u>Sample:</u> small sample size, no power analysis<br><br><u>Methodological quality:</u> no blinding, only baseline values of endpoints reported, no information on further baseline values or comparability between groups, no information on method of randomization<br><br><u>Compliance:</u> no information on compliance and dropout<br><br><u>Report quality:</u> very poor report quality (e.g., no further details on cancer type/stages, age of subjects, recruitment period and time of intervention in relation to RCTx), no explanation of results in the discussion, no conflict-of-interest statement
Choi et al. (2019)	RCT	RoB<br>Randomised assignment: low<br>Deviations from the intended interventions: high<br>Missing outcome data: low<br>Measurement of the outcome: some concerns<br>Selection of the reported result: some concerns<br><br>Overall risk of bias: high	PRO:<br><u>Sample:</u> large sample size according to power analysis<br><br><u>Methodological quality:</u> ethics vote, double blinding, groups comparable to baseline for given characteristics<br><br><u>Statistical analysis:</u> detailed statistics, correction for multiple testing<br><br><u>Report quality:</u> good report quality<br><br>CONTRA:<br><u>Methodological quality:</u> few baseline characteristics given<br><br><u>Statistical analysis:</u> no ITT<br><br><u>Compliance:</u> dropout rate 17.5% (A: 22.4%, 8.2% due to 'protocol violation', B: 12.5%, without 'protocol violation'), little information on compliance

			Report quality: no data on patient recruitment period, little information on blinding, no discussion on differences in PSA change and progression over time
Delavarian et al. (2019)	RCT	RoB<br>Randomised assignment: low<br>Deviations from the intended interventions: high<br>Missing outcome data: some concerns<br>Measurement of the outcome: some concerns<br>Selection of the reported result: some concerns<br><br>Overall risk of bias: high	PRO:<br>Methodological quality: ethics vote, baseline characteristics comparable, p-values given (but few), double blinding<br><br>Report quality: reasons for dropout given<br><br>CONTRA:<br>Sample: small sample size, no power analysis<br><br>Statistical analysis: no ITT<br><br>Report quality: little information on blinding, objectives of study (primary/second outcomes) not clearly stated, very superficial report, low report quality, few baseline characteristics given, no tumor stages, no data on period of patient recruitment
Fardad et al. (2022)	RCT	RoB<br>Randomised assignment: some concerns<br>Deviations from the intended interventions: some concerns<br>Missing outcome data: some concerns<br>Measurement of the outcome: some concerns<br>Selection of the reported result: high<br><br>Overall risk of bias: high	PRO:<br>Methodological quality: ethics vote, double blinding<br><br>Compliance: low dropout (5.3%)<br><br>CONTRA:<br>Sample: few subjects per group, no power analysis<br><br>Methodological quality: few baseline characteristics collected, different ways of applying the interventions (spray, gel, mouth rinse), thus indicated double blinding questionable; little information on how randomization was conducted and how blinding was ensured<br><br>Statistical analysis: no ITT<br><br>Compliance: little concrete information on reasons for dropout, slightly higher in B<br><br>Report quality: results over time, mostly graphical (individual values cannot be read out exactly); no indication of standard deviations or t-values; no analysis over time; only individual time points with results are reported, unclear whether further time points are statistically different, no data on period of patient recruitment, no conflict-of-interest statement

Gunther et al. (2022)	RCT	RoB<br>Randomised assignment: Some concerns<br>Deviations from the intended interventions: low<br>Missing outcome data: low<br>Measurement of the outcome: some concerns<br>Selection of the reported result: low<br><br>Overall risk of bias: Some concerns	PRO:<br>Methodological quality: ethics vote, power analysis, double blinded (patients, care providers, research team), trial protocol available<br><br>Statistical analysis: ITT<br><br>Report quality: detailed information on blinding<br><br>CONTRA:<br>Sample: very small sample size (early termination of study because only 1 of the first 15 patients reached pCR)<br><br>Methodological quality: sign. difference in age of patients (A: 69, B: 50, p=0.02), conflict of interest<br><br>Compliance: low compliance: dose adjustments/discontinuations especially in intervention group (46.7%) with already small group size (2 dose reductions during RCTx, 4 discontinuations and 1 dose reduction during maintenance therapy)<br><br>Report quality: discrepancies from treatment discontinuations listed in flowchart and mentioned in text, many missing laboratory values
Hejazi et al. (2013 and 2016)	RCT	RoB<br>Randomised assignment: low<br>Deviations from the intended interventions: low<br>Missing outcome data: low<br>Measurement of the outcome: some concerns<br>Selection of the reported result: high<br><br>Overall risk of bias: high	PRO:<br>Methodological quality: ethics vote, double blinding, no baseline differences regarding QoL<br><br>Compliance: high compliance, acceptable dropout (A: 10%, B: 15%)<br><br>Statistical analysis: ITT<br><br>CONTRA:<br>Sample: small sample, no power analysis (pilot trial)<br><br>Methodological quality: group differences from baseline (total antioxidant capacity; p = 0.045)<br><br>Report quality: little information on blinding, other endpoints calculated than described in study protocol (primary endpoints PFS and PSA after one year announced, but only QoL and PSA value after 3 months reported), poor report quality, no conflict-of-interest statement

Howells et al. (2019)	RCT	RoB<br>Randomised assignment: high<br>Deviations from the intended interventions: some concerns<br>Missing outcome data: low<br>Measurement of the outcome: some concerns<br>Selection of the reported result: low<br><br>Overall risk of bias: high	PRO:<br>Methodological quality: ethics vote, trial protocol available<br><br>Statistical analysis: ITT (efficacy outcomes) and PP analysis<br><br>CONTRA:<br>Sample: small sample, no power analysis<br><br>Methodological quality: no blinding, no p-values given for baseline values, age and performance status comparable according to data, no information on gender of participants, no information on randomization<br><br>Compliance: low compliance in curcumin group (16.7% excluded due to protocol violation/missing curcumin intake/low curcuminoid plasma levels)<br><br>Report quality: low report quality, partly missing data (e.g. PFS and OS for ITT, PP and without surgical intervention calculated according to report, but ITT not shown in diagram), no p-values for AEs given, study periods do not match information in protocol, no information how side effects were recorded
Kia et al. (2021)	RCT	RoB<br>Randomised assignment: low<br>Deviations from the intended interventions: low<br>Missing outcome data: low<br>Measurement of the outcome: some concerns<br>Selection of the reported result: some concerns<br><br>Overall risk of bias: some concerns	PRO:<br>Methodological quality: ethics vote, double blinding, power analysis, baseline characteristics comparable<br><br>Dropout/Compliance: no dropout (or no information on dropout)<br><br>Statistical analysis: correction of multiple testing<br><br>Report quality: detailed information on randomization and assurance of blinding at baseline<br><br>CONTRA:<br>Report quality: no information on recruitment period, compliance, dropout or AEs - questionable given the relatively long study period; differences in results very small - unclear if clinically relevant (no indication of effect sizes); announcement of an ANOVA whose results are not reported, instead only report of T-tests
Kumar et al. (2016)	RCT	RoB<br>Randomised assignment: Some concerns<br>Deviations from the intended interventions: high<br>Missing outcome data: high<br>Measurement of the outcome: some concerns<br>Selection of the reported result: Some concerns	PRO:<br>Methodological quality: ethics vote<br><br>CONTRA:<br>Sample: small sample, no power analysis

		Overall risk of bias: high	<u>Methodological quality:</u> baseline characteristics comparable, but p-values not reported for all characteristics, no blinding, no placebo, endpoints only imprecisely stated<br><br><u>Statistical analysis:</u> no ITT, statistical methods unclear, unclear which subgroup analyses were done and which prognostic indicators are meant, p-values are only partially reported and can only be taken from the report, tabular presentation without this information<br><br><u>Dropout/Compliance:</u> high dropout due to non-compliance, dropout not reported, only to be inferred from diagram<br><br><u>Report quality:</u> poor report quality, partly difficult to understand due to grammatical and typing errors, errors in reported results
Mansourian et al. (2015)	RCT	RoB<br>Randomised assignment: some concerns<br>Deviations from the intended interventions: low<br>Missing outcome data: low<br>Measurement of the outcome: some concerns<br>Selection of the reported result: some concerns<br><br>Overall risk of bias: some concerns	PRO:<br><u>Methodological quality:</u> ethics vote, double blinding<br><br><u>Dropout/Compliance:</u> no dropout (or no information on dropout)<br><br>CONTRA:<br><u>Sample:</u> small sample, no power analysis<br><br><u>Methodological quality:</u> possible group differences from baseline cannot be ruled out, little information on baseline characteristics (only sex, age, and RTx dose)<br><br><u>Report quality:</u> little information on blinding, poor report quality (e.g., no data on how cancer types are distributed between groups, no data on dropout and compliance)
Najafizade et al. (2023)	RCT	RoB<br>Randomised assignment: some concerns<br>Deviations from the intended interventions: low<br>Missing outcome data: low<br>Measurement of the outcome: some concerns<br>Selection of the reported result: some concerns<br><br>Overall risk of bias: some concerns	PRO:<br><u>Sample:</u> large sample size according to power analysis<br><br><u>Methodological quality:</u> ethics vote, baseline values comparable, double blinding<br><br><u>Dropout/Compliance:</u> no dropout (or no information on dropout)<br><br>CONTRA:<br><u>Sample:</u> small sample<br><br><u>Methodological quality:</u> few baseline values given, no information on method and ensuring of blinding, no information on dropout or compliance<br><br><u>Statistical analysis:</u> very simple statistics, only one p-value given for all intestinal side effects and different degrees of intestinal disorders

			Report quality: scarce report with minor errors, information on time course confusing (curcumin administration in relation to RCTx ; start of RCTx referred to as baseline, but demographic data presumably collected before)
Nakao and Ueno (2021)	RCT	RoB<br>Randomised assignment: low<br>Deviations from the intended interventions: high<br>Missing outcome data: some concerns<br>Measurement of the outcome: high<br>Selection of the reported result: some concerns<br><br>Overall risk of bias: high	PRO:<br>Methodological quality: ethics vote, double blinding<br><br>Compliance: relatively low dropout overall (7.4%), no dropout at all in placebo and turmeric group, assessment of compliance (correct administration, impression of product and side effects)<br><br>CONTRA:<br>Sample: extremely small samples due to division into 5 arms, no power analysis (pilot trial)<br><br>Methodological quality: little information on blinding, no p-values for baseline values given, little information on comparability of groups (according to report no sign. difference in radiation dose), but apparent differences in gender distribution and other parameters, comparability can be doubted, individual arms difficult to evaluate due to missing data on mean values, SD and p-values<br><br>Statistical analysis: no ITT<br><br>Report quality: low report quality, overall rather related to propolis
Panahi et al. (2014)	RCT	RoB<br>Randomised assignment: some concerns<br>Deviations from the intended interventions: high<br>Missing outcome data: some concerns<br>Measurement of the outcome: some concerns<br>Selection of the reported result: some concerns<br><br>Overall risk of bias: high	PRO:<br>Sample: large sample size<br><br>Methodological quality: ethics vote, double blinding, matched groups in terms of baseline characteristics, baseline characteristics comparable for given values<br><br>Dropout/Compliance: dropout without sign. difference in both groups ($p>0.05$)<br><br>CONTRA:<br>Methodological quality: no information on method of randomization and assurance of blinding, no power analysis; no indication of comparability of outcome parameters at baseline, QoL scores are descriptively different (A 41.67, B 69.69)<br><br>Dropout/Compliance: high dropout in both groups (reason lost to follow-up/non-appearance for final examination and blood sampling)<br><br>Statistical analysis: no ITT

			Report quality: very concise report, in particular little information on methodology, no information on recruitment period
Panahi et al. (2021)a	RCT	RoB<br>Randomised assignment: high<br>Deviations from the intended interventions: high<br>Missing outcome data: some concerns<br>Measurement of the outcome: some concerns<br>Selection of the reported result: some concerns<br><br>Overall risk of bias: high	PRO:<br>Methodological quality: ethics vote, power analysis, double blinding<br><br>CONTRA:<br>Methodological quality: variance in baseline values of ESR ($p=0.012$) and TNF-α ($p=0.009$), baseline values compared only by age, sex and laboratory parameters collected, no other characteristics, already before intervention differences in QoL scales between groups (in curcumin higher functionality, more symptoms), conflict of interest<br><br>Compliance: dropout > 5%, slightly higher in curcumin than in placebo, no reasons given<br><br>Statistical analysis: no ITT<br><br>Report quality: no information provided on randomization and ensuring of blinding, no recruitment period provided
Panahi et al. (2021)b	RCT	RoB<br>Randomised assignment: low<br>Deviations from the intended interventions: some concerns<br>Missing outcome data: low<br>Measurement of the outcome: some concerns<br>Selection of the reported result: some concerns<br><br>Overall risk of bias: some concerns	PRO:<br>Methodological quality: ethics vote, double blinding, groups matched for baseline demographics, baseline values for given patient characteristics and hematologic/biochemical parameters comparable<br><br>Dropout/Compliance: low dropout<br><br>Statistical analysis: testing for normal distribution, baseline values as covariates in the ANCOVA<br><br>Report quality: detailed information on randomization and blinding (researchers and patients)<br><br>CONTRA:<br>Methodological quality: no power analysis, conflict of interest, no p-values given for all baseline characteristics, significant difference in baseline values for 2 QoL items with higher values in intervention group (recreation and swallowing score)<br><br>Statistical analysis: no ITT analysis<br><br>Report quality: no reasons for dropout given, no explanation on the differences of the used scales for QoL

Passildas-Jahanmohan et al. (2021)	RCT	RoB<br>Randomised assignment: some concerns<br>Deviations from the intended interventions: low<br>Missing outcome data: low<br>Measurement of the outcome: some concerns<br>Selection of the reported result: some concerns<br><br>Overall risk of bias: some concerns	PRO:<br><u>Methodological quality:</u> ethics vote, baseline characteristics comparable, multicenter, double blinding<br><br><u>Compliance:</u> good compliance, has been verified<br><br><u>Statistical analysis:</u> modified ITT analysis (patients who received at least one treatment)<br><br>CONTRA:<br><u>Sample:</u> small sample size due to early study dropout, no power analysis<br><br><u>Methodological quality:</u> little information on methodology, randomization (stratified randomization by center, age, and number of lesions), and assurance of blinding, no conflict-of-interest statement
Patil et al. (2015)	RCT	RoB<br>Randomised assignment: high<br>Deviations from the intended interventions: some concerns<br>Missing outcome data: low<br>Measurement of the outcome: some concerns<br>Selection of the reported result: some concerns<br><br>Overall risk of bias: high	PRO:<br><u>Methodological quality:</u> ethics vote<br><br><u>Dropout/Compliance:</u> no dropout (or no information on dropout)<br><br>CONTRA:<br><u>Sample:</u> very small sample, no power analysis (pilot trial)<br><br><u>Methodological quality:</u> little information given on randomization, no blinding, no information on comparability of groups, hardly any baseline characteristics given, no p-values, numerical values in results table questionable<br><br><u>Dropout/Compliance:</u> no information on dropout or compliance<br><br><u>Statistical analysis:</u> no individual comparisons given - thus no information on which time points actually differ between groups (insufficient statistical analysis)<br><br><u>Report quality:</u> very concise report with little contextual information
Ramezani et al. (2023)	RCT	RoB<br>Randomised assignment: some concerns<br>Deviations from the intended interventions: high<br>Missing outcome data: some concerns<br>Measurement of the outcome: some concerns<br>Selection of the reported result: some concerns<br><br>Overall risk of bias: high	PRO:<br><u>Methodological quality:</u> ethics vote, baseline characteristics comparable<br><br><u>Dropout/Compliance:</u> dropout evenly distributed<br><br><u>Statistical analysis:</u> correction for multiple testing<br><br>CONTRA:<br><u>Sample:</u> small sample, no power analysis (pilot trial)

			Methodological quality: no double blinding (only assessor of clinical symptoms blinded), different frequency of use of mouthwash and gel capsules, no information on how often acetaminophen was needed<br><br>Dropout/Compliance: high dropout with small group size, no specific reasons given why intervention was discontinued<br><br>Statistical analysis: no ITT, statistical data partly unclear or can only be taken from the text; for group comparisons, p-value of ANOVA is given, no detailed information on post-hoc analyses between individual groups, results therefore partly not interpretable, no mean values for WHO or NRS, only shown in graph and mean difference in table<br><br>Report quality: report with minor errors
Rao et al. (2014)	RCT	RoB<br>Randomised assignment: low<br>Deviations from the intended interventions: some concerns<br>Missing outcome data: low<br>Measurement of the outcome: high<br>Selection of the reported result: some concerns<br><br>Overall risk of bias: high	PRO:<br>Methodological quality: ethics vote, active control<br><br>Sample: adequate sample according to power analysis<br><br>Dropout/Compliance: low dropout (A: 2.5%, B: 0%)<br><br>CONTRA:<br>Methodological quality: curcumin arm has to rinse mouth much more frequently than control arm - higher frequency in curcumin could have influence on better results, independent of active ingredient, no double blinding, no information how blinding of investigators could be ensured, no information on comparability of groups, no p-values given for baseline values<br><br>Statistical analysis: no ITT<br><br>Report quality: poor report quality (e.g., in some cases numbers can only be taken from graphs), inadequate presentation of results, no transparency in analyses due to missing values
Ryan et al. (2013)	RCT	RoB<br>Randomised assignment: low<br>Deviations from the intended interventions: high<br>Missing outcome data: low<br>Measurement of the outcome: some concerns<br>Selection of the reported result: low<br><br>Overall risk of bias: high	PRO:<br>Methodological quality: ethics vote, double blinded with control (A: 5/14 [35.7%] of patients think in curcumin arm, B: 2/16 [12.5%] of patients think in placebo arm), recording of expected and current pain at baseline<br><br>Dropout/Compliance: high comparable compliance rates (mean [SD]: A: 96.6% [6.6%], B: 98.4% [3.2%]; p = 0.344)<br><br>CONTRA:<br>Sample: very small sample, no power analysis

			<u>Dropout/Compliance:</u> dropout rate: A: 18%, B: 11%<br><br><u>Statistical analysis:</u> no ITT, no control of multiple testing<br><br><u>Report quality:</u> little information on blinding, no conflict-of-interest statement
Ryan Wolf et al. (2017)	RCT	RoB<br>Randomised assignment: some concerns<br>Deviations from the intended interventions: low<br>Missing outcome data: low<br>Measurement of the outcome: some concerns<br>Selection of the reported result: some concerns<br><br>Overall risk of bias: some concerns	PRO:<br><u>Sample:</u> large sample, multicenter, power analysis<br><br><u>Methodological quality:</u> double blinded (also blinded assessors of photo documentation regarding RDS and wet desquamation to study evaluation by digital photos), baseline characteristics comparable, but variations in ER/PR (estrogen receptor/progesterone receptor) status and CTx before RTx (in curcumin group fewer ER tumors and patients with CTx before RTx), recording of side effects using SI, detailed explanation of measurement tools<br><br><u>Dropout/Compliance:</u> compliance checked by pill counting (no sign. differences), extensively reported on dropout and reasons per group<br><br><u>Statistical analysis:</u> ITT for primary analysis<br><br>CONTRA:<br><u>Methodological quality:</u> "standard care" for dermatitis - not described in detail (discussion mentions how variable between centers)<br><br><u>Dropout/Compliance:</u> high dropout (16.8%), higher in curcumin than placebo<br><br><u>Statistical analysis:</u> PP analysis (2nd to 5th endpoint)<br><br><u>Report quality:</u> only graphical presentation of results of primary analysis, table with exact values over time would be more transparent, no representation of symptoms over time
Ryan Wolf et al. (2020)	RCT	RoB<br>Randomised assignment: low<br>Deviations from the intended interventions: high<br>Missing outcome data: low<br>Measurement of the outcome: some concerns<br>Selection of the reported result: some concerns<br><br>Overall risk of bias: high	PRO:<br><u>Sample:</u> large sample, power analysis, analysis of blinding and acceptability<br><br><u>Methodological quality:</u> baseline characteristics comparable, “standard care” survey<br><br><u>Dropout/Compliance:</u> compliance verified and comparable (self-report), detailed report of reasons for dropout<br><br><u>Statistical analysis:</u> sensitivity analysis with multiple imputation<br><br>CONTRA:

			<u>Methodological quality:</u> no double blinding (only patients and reviewers of photos), HPR Plus™ with different color and consistency than curcumin/placebo (but blinding presumed intact according to analysis), no indication of the distribution of breast field separation between groups, conflict of interest<br><br><u>Dropout/Compliance:</u> dropout total 10.1%<br><br><u>Statistical analysis:</u> PP analysis
Saadipoor et al. (2018)	RCT	RoB<br>Randomised assignment: low<br>Deviations from the intended interventions: low<br>Missing outcome data: low<br>Measurement of the outcome: some concerns<br>Selection of the reported result: low<br><br>Overall risk of bias: some concerns	PRO:<br><u>Methodological quality:</u> ethics vote, power analysis, triple blinding (patients, health care providers, data collectors, outcome assessors)<br><br><u>Dropout/Compliance:</u> hardly any dropout (in control group one RTx discontinuation due to grade 3 radiation-induced proctitis/GI bleeding)<br><br><u>Statistical analysis:</u> ITT in 1st to 3rd<br><br><u>Report quality:</u> detailed information on blinding<br><br>CONTRA:<br><u>Methodological quality:</u> no data on comparability of groups/no p-values given for baseline characteristics, no indication of AEs<br><br><u>Report quality:</u> no precise information on duration of RTx and curcumin administration
Saghatelyan et al. (2020)	RCT	RoB<br>Randomised assignment: low<br>Deviations from the intended interventions: low<br>Missing outcome data: Some concerns<br>Measurement of the outcome: some concerns<br>Selection of the reported result: some concerns<br><br>Overall risk of bias: Some concerns	PRO:<br><u>Methodological quality:</u> ethics vote, double blinding, power analysis, baseline characteristics comparable<br><br><u>Dropout/Compliance:</u> detailed evaluation of dropout reasons, examination of compliance<br><br><u>Statistical analysis:</u> ITT for time-to-event outcomes<br><br><u>Report quality:</u> detailed information on blinding<br><br>CONTRA:<br><u>Methodological quality:</u> conflict of interest<br><br><u>Report quality:</u> diverse statistical analyses with some reported in an unclear manner, inconsistencies between table and text regarding AEs

Sandoughdaran et al. (2021)	RCT	RoB<br>Randomised assignment: low<br>Deviations from the intended interventions: high<br>Missing outcome data: low<br>Measurement of the outcome: some concerns<br>Selection of the reported result: some concerns<br><br>Overall risk of bias: high	PRO:<br>Methodological quality: ethics vote, double blinding, ensuring of blinding before treatment initiation, baseline characteristics comparable<br><br>Statistical analysis: ITT analysis for secondary endpoints<br><br>Report quality: detailed information on blinding, detailed randomization information (internet-generated by person not involved in trial, stratified by chemotherapy [gemcitabine/cisplatin vs. gemcitabine/carboplatin] and treatment center)<br><br>CONTRA:<br>Methodological quality: few baseline characteristics - no gender distribution given<br><br>Sample: small sample size, no power analysis (pilot trial)<br><br>Statistical analysis: PP analysis for primary endpoint<br><br>Dropout/Compliance: high dropout especially in curcumin group
Santosa et al. (2022)	RCT	RoB<br>Randomised assignment: low<br>Deviations from the intended interventions: high<br>Missing outcome data: some concerns<br>Measurement of the outcome: some concerns<br>Selection of the reported result: low<br><br>Overall risk of bias: high	PRO:<br>Methodological quality: ethics vote, power analysis, baseline characteristics comparable<br><br>Report quality: detailed listing of dropout reasons<br><br>CONTRA:<br>Sample: small sample size (pilot trial)<br><br>Methodological quality: no double blinding (only patients blinded)<br><br>Statistical analysis: no ITT<br><br>Dropout/Compliance: very high dropout (24.2% deceased overall, equally distributed in both groups)<br><br>Report quality: scarce baseline information, only age and clinical parameters (no context to patient behavior or history), inconsistencies between reported values in text and associated table, hardly any information from which time point the reported data originated or which data were collected at the other time points

Shah et al. (2020)	RCT	RoB<br>Randomised assignment: low<br>Deviations from the intended interventions: low<br>Missing outcome data: high<br>Measurement of the outcome: high<br>Selection of the reported result: some concerns<br><br>Overall risk of bias: high	PRO:<br>Methodological quality: ethics vote, baseline characteristics comparable, testing of compliance, triple blinded (participants, principal investigator, statistician)<br><br>Statistical analysis: modified ITT (patients with at least one follow-up)<br><br>Report quality: detailed information on blinding<br><br>CONTRA:<br>Methodological quality: no power analysis (pilot trial), no reasons for dropout given, very confusing analysis of results with numerous analyses comparing individual study periods with each other, a lot of missing outcome data at the late examination periods (at last 75% missing, PP analysis only with 17 of the 68 patients included in the analysis [25%])<br><br>Statistical analysis: mITT only used in one table to present scores, other analyses PP (not possible due to missing data)
Soni et al. (2021)	RCT	RoB<br>Randomised assignment: some concerns<br>Deviations from the intended interventions: low<br>Missing outcome data: low<br>Measurement of the outcome: some concerns<br>Selection of the reported result: some concerns<br><br>Overall risk of bias: some concerns	PRO:<br>Methodological quality: power analysis, double blinding, ITT<br><br>Dropout/Compliance: no dropout (or no information on dropout)<br><br>CONTRA:<br>Methodological quality: baseline characteristics for sex, age, performance status, and primary tumor reportedly comparable, but no statistical comparison given (age apparently differs), little information on randomization and assurance of blinding, BTF capsules in curcumin groups have to be taken differently often (blinding still ensured?), no control of compliance, additional intake of protein-rich powder - amount not standardized, descriptive age difference between groups, curcumin (lower dose) is on average 5 to 6 years younger than other groups<br><br>Report quality: no data on patient recruitment period, no indication of AEs or other events, although there were patients whose therapy had to be interrupted or were hospitalized, no information on dropout, no indication of exactly which score was used for mucositis assessment
Talakesh et al. (2022)	RCT	RoB<br>Randomised assignment: some concerns<br>Deviations from the intended interventions: low<br>Missing outcome data: low<br>Measurement of the outcome: some concerns<br>Selection of the reported result: some concerns<br><br>Overall risk of bias: some concerns	PRO:<br>Methodological quality: ethics vote, detailed information on blinding, triple blinded (patients, oncologist, statistician), power analysis, baseline characteristics comparable<br><br>Dropout/Compliance: no dropout (or no information on dropout)<br><br>Report quality: detailed information on blinding

			CONTRA:<br><u>Sample:</u> small sample size<br><br><u>Methodological quality:</u> conflict of interest<br><br><u>Dropout/Compliance:</u> no data on compliance/possible dropout<br><br><u>Report quality:</u> no precise indication of how additional aloe vera gel was used, no indication of AEs
Thambamroong et al. (2022)	RCT	RoB<br>Randomised assignment: some concerns<br>Deviations from the intended interventions: low<br>Missing outcome data: low<br>Measurement of the outcome: some concerns<br>Selection of the reported result: some concerns<br><br>Overall risk of bias: some concerns	PRO:<br><u>Methodological quality:</u> ethics vote, power analysis, randomization stratified by metastasis status, double blinding<br><br><u>Statistical analysis:</u> ITT analysis<br><br><u>Dropout/Compliance:</u> "relatively good compliance" in both groups, dropout the same in both groups (both due to gastric tube blockage at week 7), no dose reductions/interruptions observed<br><br><u>Report quality:</u> detailed information on blinding<br><br>CONTRA:<br><u>Sample:</u> very small sample size<br><br><u>Methodological quality:</u> baseline characteristics without p-values (especially control group seems to be older in range), no data on gender of subjects, placebo containing probiotics<br><br><u>Dropout/Compliance:</u> dropout 10% (with very small sample size, but same in both groups)<br><br><u>Report quality:</u> no information on duration of the recruitment period
Thomas et al. (2023)	RCT	RoB<br>Randomised assignment: low<br>Deviations from the intended interventions: high<br>Missing outcome data: low<br>Measurement of the outcome: some concerns<br>Selection of the reported result: some concerns<br><br>Overall risk of bias: high	PRO:<br><u>Methodological quality:</u> ethics vote, power analysis, baseline characteristics comparable<br><br><u>Sample:</u> large sample<br><br>CONTRA:<br><u>Methodological quality:</u> no blinding - partial patient-reported outcomes, no comparison with placebo, high dropout at week 7 - no ITT, treatment very complex with frequent mouth rinsing especially intervention group; outcome variables for week 7 only with about half of patients, as others completed RTx at week 6 - unclear if groups still comparable and comparison

	between baseline and week 7 meaningful; patients chose envelope themselves - possibility of interaction at randomization especially due to open design, no information on AEs<br><br>Statistical analysis: no differential values given (e.g. baseline - week 7), no comparisons between weeks, only indication of differential change over time

AEs: adverse events, CTx: chemotherapy, ITT: Intention-to-treat, mITT: modified intention-to-treat, pCR: pathological complete response, PFS: progression-free survival, PP: Per Protocol, PSA: prostate specific antigen, QoL: quality of life, RCT: Randomized controlled trial, RCTx: radiochemotherapy, RTx: radiotherapy
