# Supplementary material for: Curcumin as a complementary treatment in oncological therapy: a systematic review
Source: Eur J Clin Pharmacol. 2024 Oct 19;81(1):1–33. doi: 10.1007/s00228-024-03764-9 (PMC11695395; doi:10.1007/s00228-024-03764-9)
Supplement: Supplementary file 2 — Supplementary file2 (PDF 189 KB) [file 228_2024_3764_MOESM2_ESM.pdf]

Supplementary Information 2

Curcumin as a Complementary Treatment in Oncological Therapy: A Systematic Review

Supportive Care in Cancer

Lisa C. Gutsche, Jennifer Dörfler, MSc., Prof. Dr. med. Jutta Hübner

Corresponding Author: Lisa C. Gutsche, Klinik für Innere Medizin II, Hämatologie und Internistische Onkologie, Universitätsklinikum Jena, Am Klinikum 1, 07747 Jena, Germany, lisacgutsche@gmail.com

Table S2: Excluded studies

References	Study Type	Year	Title	Reason for exclusion
J. K. Acharya	RCT	2022	Efficacy of honey, triphala and turmeric mouthwash on mucosal pain management in palliative care patients	Multipreparation
H. Ansari, A. Hajigholami and D. Abbasi	RCT	2023	The Protective Effect of Curcumin on Hepatic Function in Cancer Patients Receiving Taxane-Based Chemotherapy: a Randomized Controlled Clinical Trial	No patient-relevant outcomes
S. A. Ara, J. Mudda, A. Lingappa, P. Rao and S. Zakaullah	RCT	2018	Efficacy of curcumin in oral submucous fibrosis - a randomized controlled clinical trial	Precancerous lesions
D. A. C. Arantes, A. C. G. da Silva, M. C. Valadares, N. M. A. Freitas, E. M. Lima, A. C. de Oliveira, et al.	RCT	2021	Safety and efficacy of a mucoadhesive phytomedication containing curcuminoids and Bidens pilosa L. extract in the prevention and treatment of radiochemotherapy-induced oral mucositis: A triple-blind, randomized, placebo-controlled, clinical trial	Multipreparation
C. Ariyasriwatana, N. Phoolcharoen, S. Oranratanaphan and P. Worasethsin	RCT	2022	Efficacy of Curcuminoids in Managing Postoperative Pain after Total Laparoscopic Hysterectomy: A Randomized Controlled, Open-Label Trial	Less than 80% cancer patients

K. Ataei-Almanghadim, A. Farshbaf-Khalili, A. R. Ostadrahimi, E. Shaseb, M. Mirghafourvand	RCT	2019	The effect of oral capsule curcumin and vitamin E in comparison to placebo on serum levels of some antioxidants and inflammatory factors in postmenopausal women: a randomized, triple-controlled clinical trial	Less than 80% cancer patients
M. A. Avila-Galvez, A. Gonzalez-Sarrias, F. Martinez-Diaz, B. Abellan, A. J. Martinez-Torrano, A. J. Fernandez-Lopez, et al.	RCT	2021	Disposition of Dietary Polyphenols in Breast Cancer Patients' Tumors, and Their Associated Anticancer Activity: The Particular Case of Curcumin	No patient-relevant outcomes
R. de Cassia Dias Viana Andrade, T. Azevedo Reis, L. P. Rosa, G. P. de Oliveira Santos and F. da CristinaSilva	RCT	2022	Comparative randomized trial study about the efficacy of photobiomodulation and curcumin antimicrobial photodynamic therapy as a coadjuvant treatment of oral mucositis in oncologic patients: antimicrobial, analgesic, and degree alteration effect	Curcumin with photodynamic therapy
N. Chaitanya, R. Badam, A. S. Aryasri, S. Pallarla, K. Garlpati, M. Akhila, et al.	RCT	2020	Efficacy of Improvised Topical Zinc (1%) Ora-Base on Oral Mucositis during Cancer Chemo-Radiation-A Randomized Study	Multipreparation
W. T. L. Chen, T. S. Yang, H. C. Chen, H. H. Chen, H. C. Chiang, T. C. Lin, C. H. Yeh, T. W. Ke, J. S. Chen, K. H. Hsiao and M. L. Kuo	RCT	2014	Effectiveness of a novel herbal agent MB-6 as a potential adjunct to 5-fluoracil-based chemotherapy in colorectal cancer	Multipreparation
A. L. Cheng, C. H. Hsu, J. K. Lin, M. M. Hsu, Y. F. Ho, T. S. Shen, J. Y. Ko, J. T. Lin, B. R. Lin, W. Ming-Shiang, H. S. Yu, S. H. Jee, G. S. Chen, T. M. Chen, C. A. Chen, M. K. Lai, Y. S. Pu, M. H. Pan, Y. J. Wang, C. C. Tsai and C. Y. Hsieh	RCT	2001	Phase I clinical trial of curcumin, a chemopreventive agent, in patients with high-risk or pre-malignant lesions	Precancerous lesions
A. Fani Pakdel, A. Hatami, R. Salek, A.	RCT	2021	Effects of a polyherbal formulation on the quality of life and survival of	Multipreparation

Taghizadeh-Kermani, S. A. Javadinia and A. Ghorbani			patients with common upper gastrointestinal cancers: A randomized placebo-controlled trial	
M. Farhadi, M. Bakhshandeh, B. Shafiei, A. Mahmoudzadeh and S. J. Hosseinimehr	RCT	2018	The radioprotective effects of nano-curcumin against genotoxicity induced by iodine-131 in patients with differentiated thyroid carcinoma (DTC) by micronucleus assay	No patient-relevant outcomes
W. Feize, L. Meng, L. Yanni, L. Yuan, J. Liqun, L. Tong, et al.	RCT	2017	A Randomized Controlled Study to Observe the Efficacy of External Treatment With a Traditional Chinese Medicine Herbal Ointment on Malignant Plural Effusion: Outcome Report and Design Review	Multipreparation
L. L. Fonseca, C. P. Duraes, A. S. d. S. Menezes, A. T. L. Tabosa, C. U. Barbosa, A. d. P. S. Filho, et al.	RCT	2022	Comparison between two antimicrobial photodynamic therapy protocols for oral candidiasis in patients undergoing treatment for head and neck cancer: A two-arm, single-blind clinical trial	Curcumin with photodynamic therapy
V. Ghalaut, L. Sangwan, K. Dahiya, P. Ghalaut, R. Dhankhar and R. Saharan	RCT	2012	Effect of imatinib therapy with and without turmeric powder on nitric oxide levels in chronic myeloid leukemia	No patient-relevant outcomes
T. Golombick, T. H. Diamond, V. Badmaev, A. Manoharan and R. Ramakrishna	RCT	2009	The potential role of curcumin in patients with monoclonal gammopathy of undefined significance - Its effect on paraproteinemia and the urinary N-telopeptide of type I collagen bone turnover marker	Precancerous lesions
T. Golombick, T. Diamond, A. Manoharan and R. Ramakrishna	RCT	2012	Monoclonal gammopathy of undetermined significance, smoldering multiple myeloma, and curcumin: a randomized, double-blind placebo-controlled cross-over 4g study and an open-label 8g extension study	Less than 80% cancer patients, majority precancerous lesions

Y. M. Hidayat, F. Wagey, D. Suardi, H. Susanto, B. J. Laihad and M. D. L. Tobing	RCT	2021	Analysis of Curcumin as a Radiosensitizer in Cancer Therapy with Serum Survivin Examination: Randomised Control Trial	No patient-relevant outcomes
H. Ide, S. Tokiwa, K. Sakamaki, K. Nishio, S. Isotani, S. Muto, T. Hama, H. Masuda and S. Horie	RCT	2010	Combined inhibitory effects of soy isoflavones and curcumin on the production of prostate-specific antigen	Multipreparation
E. A. De Jaeghere, S. Tuyaerts, A. M. T. Van Nuffel, A. Belmans, K. Bogaerts, R. Baiden-Amissah, et al.	RCT	2023	Pembrolizumab, radiotherapy, and an immunomodulatory five-drug cocktail in pretreated patients with persistent, recurrent, or metastatic cervical or endometrial carcinoma: results of the phase II PRIMMO study	Multipreparation
M. A. Kuriakose, K. Ramdas, B. Dey, S. Iyer, G. Rajan, K. K. Elango, A. Suresh, D. Ravindran, R. R. Kumar, R. Prathiba, S. Ramachandran, N. A. Kumar, G. Thomas, T. Somanathan, H. K. Ravindran, K. Ranganathan, S. B. Katakam, S. Parashuram, V. Jayaprakash and M. R. Pillai	RCT	2016	A randomized double-blind placebo-controlled phase iib trial of curcumin in oral leukoplakia	Precancerous lesions
C. Manfredi, L. Spirito, F. P. Calace, R. Balsamo, M. Terribile, M. Stizzo, et al.	RCT	2022	Oral Preparation of Hyaluronic Acid, Chondroitin Sulfate, Curcumin, and Quercetin (Ialuril R Soft Gels) for the Prevention of LUTS after Intravesical Chemotherapy	Multipreparation
A. F. L. Martins, C. H. Pereira, M. O. Morais, S. S. de Sousa-Neto, M. C. Valadares, N. M. A. Freitas, et al.	RCT	2023	Effects of a mucoadhesive phytomedicine (Curcuma longa L. and Bidens pilosa L.) on radiotherapy-induced oral mucositis and quality of life of patients undergoing head and neck cancer treatment: randomized clinical trial	Multipreparation
M. C. Neetha, M. G. Panchaksharappa, S. Pattabhiramasastry, N.	RCT	2020	Chemopreventive Synergism between Green Tea Extract and Curcumin in Patients with	Precancerous lesions

V. Shivaprasad and U. G. Venkatesh			Potentially Malignant Oral Disorders: A Double-blind, Randomized Preliminary Study	
P. Palatty, A. Azmidah, S. Rao, D. Jayachander, K. Thilakchand, M. Rai, R. Haniadka, P. Simon, R. Ravi, R. Jimmy, P. D'Souza, R. Fayad and M. Baliga	RCT	2014	Topical application of a sandal wood oil and turmeric based cream prevents<br><br>radiodermatitis in head and neck cancer patients undergoing external beam radiotherapy: a pilot study	Multipreparation
S. Purbadi, P. Rustamadji, A. R. Prijanti, S. M. Sekarutami, B. Sutrisna, F. D. Suyatna, et al.	RCT	2020	Biocurcumin as Radiosensitiser for Cervical Cancer Study (BRACES): A Double-Blind Randomised Placebo-Controlled Trial	Side effects of curcumin, no patient-relevant outcomes
A. Rakha, K. Rehman, M. B. Imran, M. Shahid and N. Jahan	RCT	2022	Mitigation of 131I induced oxidative stress by supplementation of turmeric and green cardamom in thyroid patients	less than 80% cancer patients, no patient-relevant outcomes
S. Rao, S. K. Hegde, M. P. Baliga-Rao, J. Lobo, P. L. Palatty, T. George and M. S. Baliga	RCT	2017	Sandalwood Oil and Turmeric-Based Cream Prevents Ionizing Radiation-Induced Dermatitis in Breast Cancer Patients: Clinical Study	Multipreparation
J. P. Redorta, F. Sanguedolce, G. S. Pardo, M. Romancik, G. Vittori, A. Minervini, et al.	RCT	2021	Multicentre International Study for the Prevention with iAluRil of Radio-induced Cystitis (MISTIC): A Randomised Controlled Study	Multipreparation
E. X. D. Santos Filho, D. A. C. Arantes, A. F. Oton Leite, A. C. Batista, E. F. d. Mendonca, R. N. Marreto, et al.	RCT	2018	Randomized clinical trial of a mucoadhesive formulation containing curcuminoids (Zingiberaceae) and Bidens pilosa Linn (Asteraceae) extract (FITOPROT) for prevention and treatment of oral mucositis - phase I study	Multipreparation
R. Thomas, M. Williams, H. Sharma, A. Chaudry and P. Bellamy	RCT	2014	A double-blind, placebo-controlled randomised trial evaluating the effect of a polyphenol-rich whole food supplement on PSA progression in men with prostate cancer - The UK NCRN Pomi-T study	Multipreparation
M. D. van Die, S. G. Williams, J. Emery, K. M. Bone, J. M. G.	RCT	2017	A Placebo-Controlled Double-Blinded Randomized Pilot Study of Combination Phytotherapy in	Multipreparation

Taylor, E. Lusk and M. V. Pirotta			Biochemically Recurrent Prostate Cancer	
H. Zahedi, M. J. Hosseinzadeh-Attar, B. Barkhidarian, S. Hosseini, M. Shadnoush, A. Sahebkar, et al.	RCT	2021	Effects of curcuminoids on inflammatory and oxidative stress biomarkers and clinical outcomes in critically ill patients: A randomized double-blind placebo-controlled trial	Less than 80% cancer patients
K. E. Zuniga, D. L. Parma, E. Munoz, M. Spaniol, M. Wargovich and A. G. Ramirez	RCT	2018	Dietary intervention among breast cancer survivors increased adherence to a Mediterranean-style, anti-inflammatory dietary pattern: the Rx for Better Breast Health Randomized Controlled Trial	Less than 80% cancer patients
